# Supplementary material for: Measuring Integrin Force Loading Rates Using a Two-Step DNA Tension Sensor
Source: J Am Chem Soc. 2024 Aug 12;146(33):23034–43. doi: 10.1021/jacs.4c03629 (PMC11345772; doi:10.1021/jacs.4c03629)
Supplement: Supplementary file 1 — ja4c03629_si_001.pdf [file ja4c03629_si_001.pdf]

Supplementary Information for

Measuring integrin force loading rates using a two-step DNA tension sensor

J. Dale Combs[1‡], Alexander K. Foote[1‡], Hiroaki Ogasawara[1], Arventh Velusamy[1], Sk Aysha Rashid[1], Joseph Nicholas Mancuso[1], and Khalid Salaita[1,2].*

‡These authors contributed equally.

1 Department of Chemistry, Emory University, Atlanta, GA 30322, USA.

2 Wallace H. Coulter Department of Biomedical Engineering, Georgia Institute of Technology and Emory University, Atlanta, GA 30322, USA.

*Correspondence should be addressed: k.salaita@emory.edu

Table of Contents

Figure S1. Optical configuration for emission resolved FRET S3
Figure S2. LR probe surface preparation step diagram. S4
Figure S3. Titration of biotinylated surfaces with different concentration of LR probe. S5
Figure S4. Selection of FRET traces by acceptor, donor photobleaching S6
Figure S5. Analysis of single molecule intensity, FRET efficiency, and survival lifetime of LR probe Cy3B S7
Figure S6. Evolution of LR probe surfaces exposed to hairpin locking strand. S8
Figure S7. Additional traces from Figure 2a showing single-step photobleaching S9
Figure S8. Force-extension curves of the LR probe as modeled by oxDNA S10
Figure S9. Identification of clean, single molecule events for further single-molecule analysis S11

SI Note 1 – LR Probe Concept S12
Table S1. Sequences of modified oligonucleotides as custom synthesized by Integrated DNA Technologies S13

Experimental and Method Sections
SI Note 2 – LR Probe Synthesis – Experimental Procedure S14
Figure S10. Synthesis of Cy3B-Biotin-Azide S17
Figure S11. LR anchor HPLC chromatograms S18
Figure S12. Synthesis and purification of cRGD-Azide S19
Figure S13. LR ligand HPLC chromatograms. S20
Figure S14. cRGD-dsDNA-biotin HPLC chromatograms. S21

SI Note 3 – Modeling of F_{shear} with variable loading rate S22
Figure S15. Results from a loading-rate dependent shearing model S23

SI Note 4 – LR Probe Surface Preparation – Experimental Procedure S24

SI Note 5 – Live Cell Imaging – Experimental Procedure S26
Figure S16. Representative loading rate trace localizations at a 10 sec. frame interval S28
Figure S17. Representative loading rate trace localizations at a 20 sec. frame interval S29
Figure S18. Representative loading rate trace localization at a 1 min. frame interval S30
Figure S19. Manually identified loading time and linear loading rate for different frame interval S31

SI Note 6 – OxDNA Modeling of the LR Probe S32
Table S2. Settings used for oxDNA modeling of LR probe force transitions S32

SI Note 7 – Automated Analysis of Single-Molecule Data S34
Figure S20. Single molecule fluorescence intensity Gaussian fitting S36
Figure S21. Distributions of the x0 and y0 values from Gaussian fits S38
Figure S22. Time trace derivation from single-molecule events S40
Figure S23. Single-molecule traces after implementing a change-point algorithm S41
Figure S24. Time traces for Cy3B (green) and FRET (red) channels for rupture events S45

SI Note 8 – Single-cell loading rate distributions S48
Figure S25. Loading rate distributions for each cell S48

SI Note 9 – ESI-MS S50

SI Note 10 – Manual and Picasso assisted analysis of single-molecule data S51

SI Note 11 – LR Probes Report Integrin-Ligand Tension S52
Figure S26. Loading rate probes report cell tension in Cy3B, A647N (640 nm excitation), and sensitized FRET fluorescence channels S54
Figure S27. LR probes report ligand specific opening and shearing at low probe density S55
Figure S28. Localizations of different LR probe anchor constructs S56

References S58

SI Figures

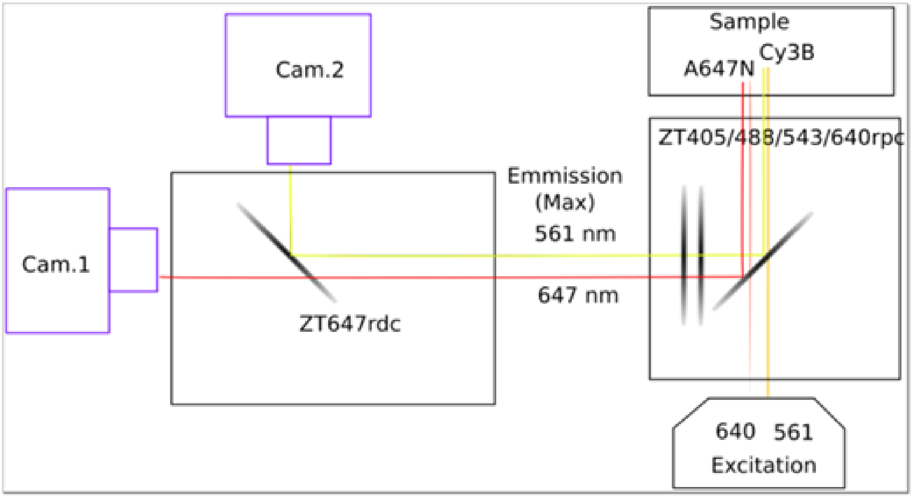

Figure S1. Optical configuration for emission resolved FRET. Laser lines of 561 and 647 nm are indicated as well as dichroic mirrors, emission filters, sample, and cameras to image Cy3B emission (Cam. 2) and A647N emission (Cam. 1).

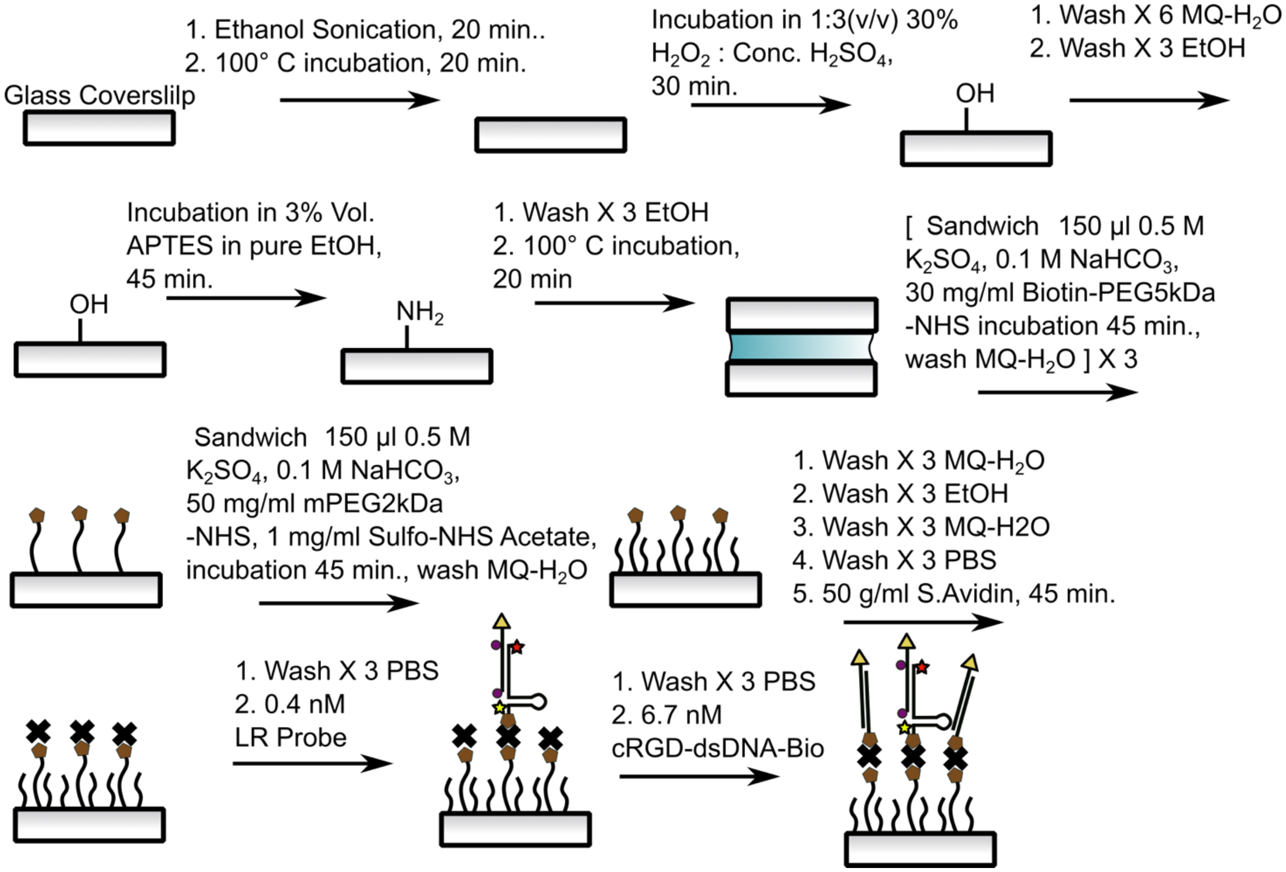

Figure S2. LR probe surface preparation step diagram. A detailed description of each of these steps can be found in **SI Note 4 – LR Probe Surface Preparation**.

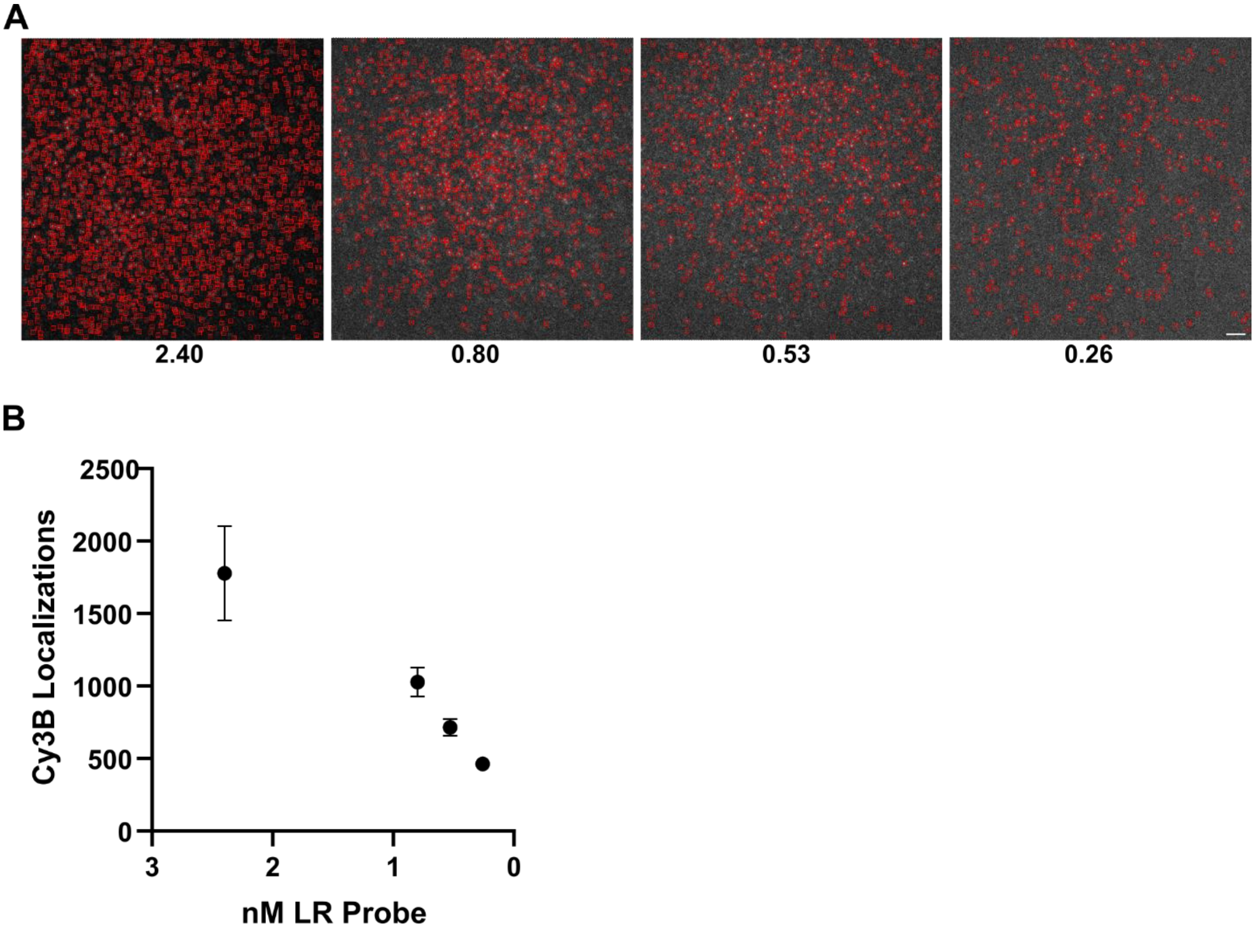

Figure S3. Titration of biotinylated surfaces with different concentration of LR probe. (A) Representative micrographs of surfaces exposed to 2.40, 0.80, 0.53, and 0.26 nM hybridized ligand and anchor strand LR probe with localizations boxed in red by Picasso software using settings of box size = 7, Min. Net Gradient: 19700.[9] (B) Plot of localizations within FOV (2982 μm^2) vs concentration of LR probe exposure for three Cy3B fluorescence micrographs of one surface per concentration. Scale bar = 3 µm.

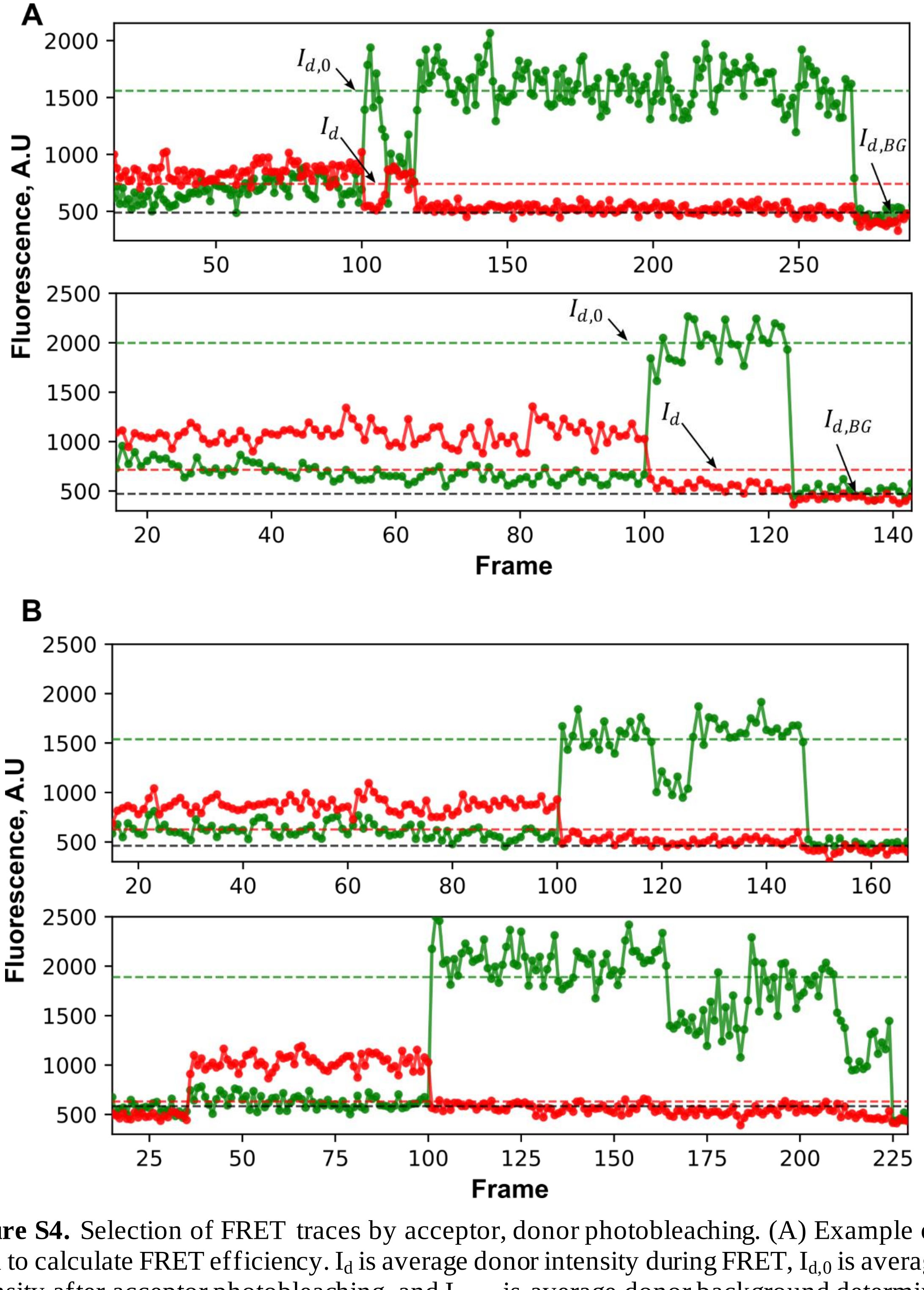

Figure S4. Selection of FRET traces by acceptor, donor photobleaching. (A) Example of traces used to calculate FRET efficiency. I_d is average donor intensity during FRET, $I_{d,0}$ is average donor intensity after acceptor photobleaching, and $I_{d,BG}$ is average donor background determined after donor photobleaching. (B) Examples of traces rejected from analysis due to Cy3B trace shifting between intensity values indicating dark states and intensities indicating more than one molecule.

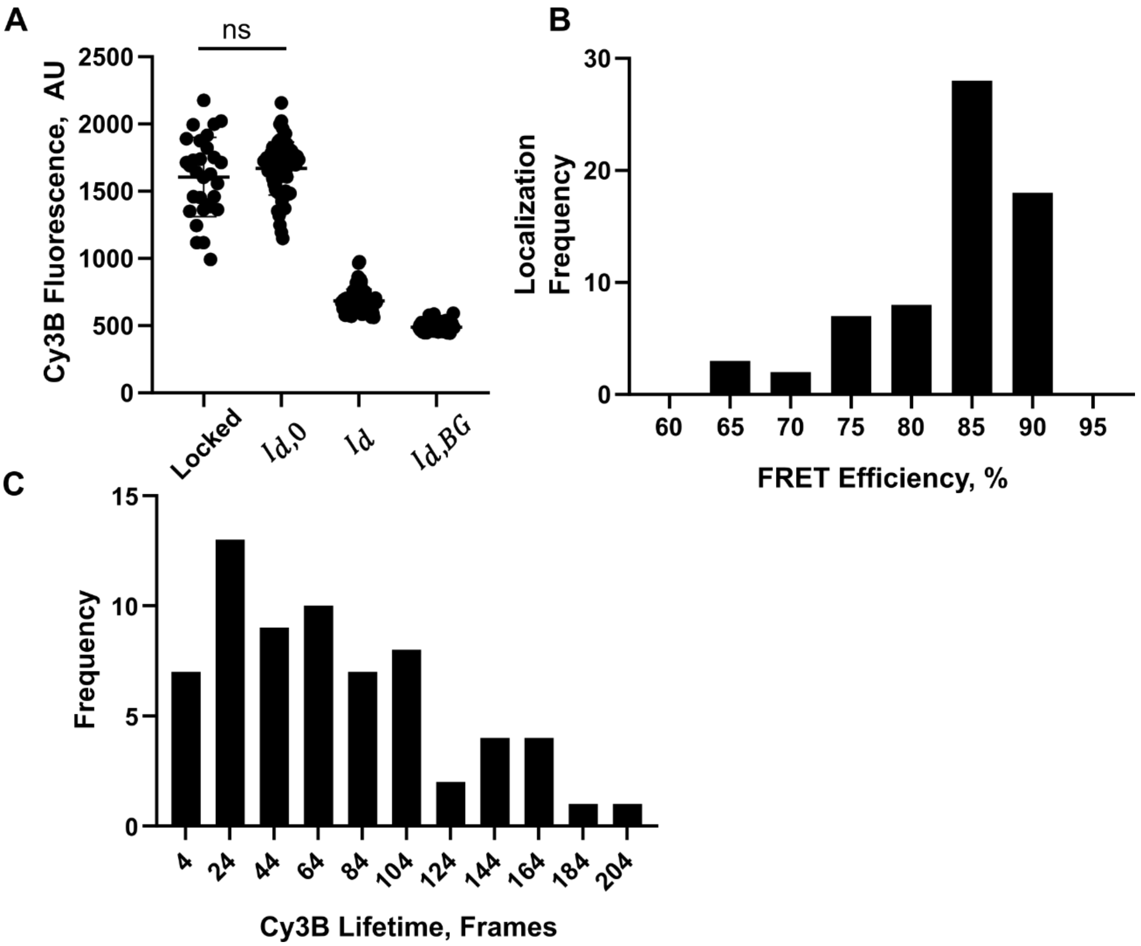

Figure S5. Analysis of single molecule intensity, FRET efficiency, and survival lifetime of LR probe Cy3B. (**A**) Fluorescence intensity of single molecule Cy3B traces upon locking strand induced opening of anchor strand hybridized to ligand strand with quencher(Locked), after single-anchor stranded acceptor photobleaching(Id0), during FRET(Id), and after Cy3B photobleaching(Id,BG). (**B**) Histogram of FRET efficiency calculated from single molecule Cy3B traces of single anchor strand. The FRET efficiency $\boldsymbol{E} = \frac{I_{d,0}-I_d}{I_{d,0}-I_{d,BG}}$ was calculated where $\boldsymbol{I_{d,0}}$ is the donor intensity after acceptor photobleaching, $\boldsymbol{I_d}$is the donor intensity during FRET, $\boldsymbol{I_{d,BG}}$ is the donor channel background intensity determined after donor photobleaching. (C) Cy3B exposure lifetime after as calculated by the number of frames where Cy3B is bright after A647N acceptor photobleaching.

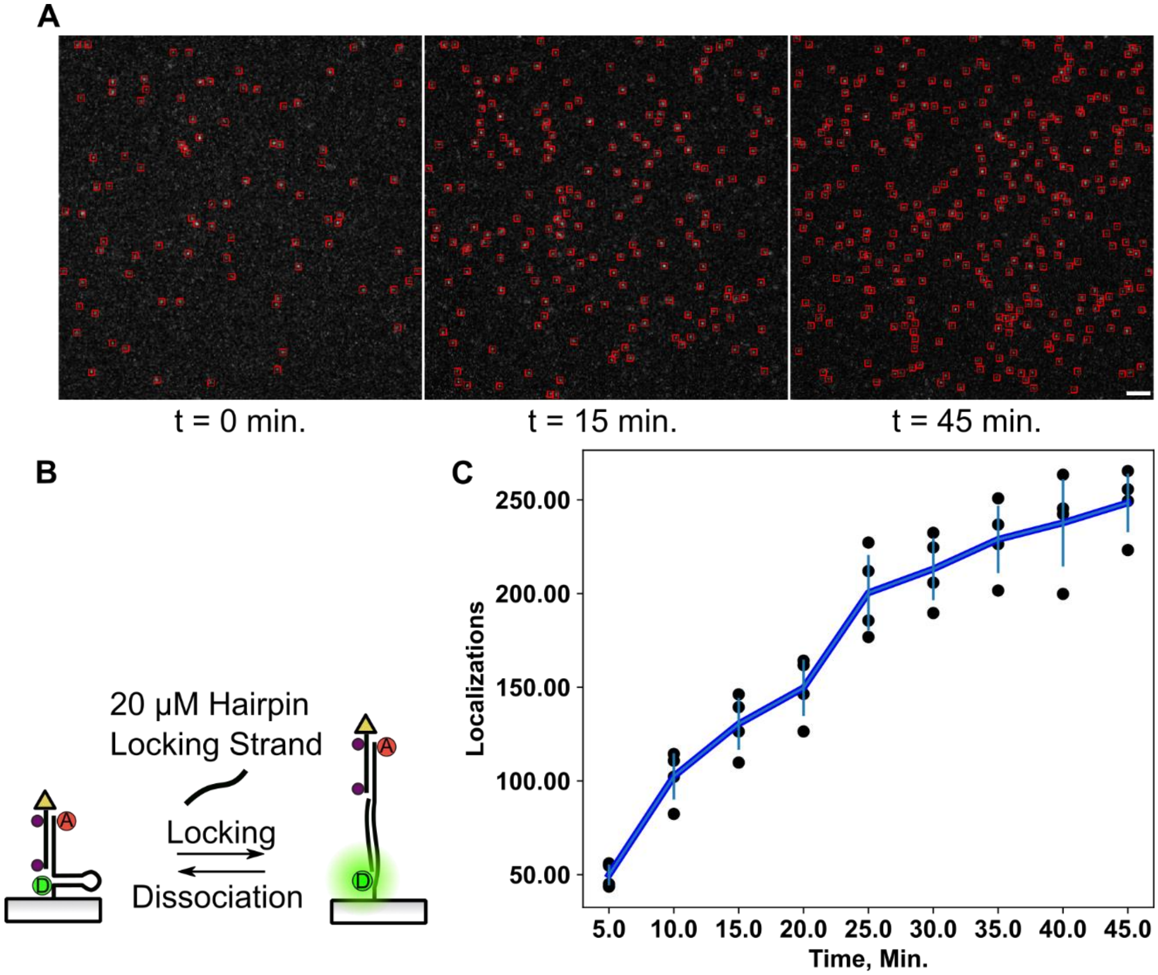

Figure S6. LR probe surfaces exposed to hairpin locking strand.
(**A**) Fluorescence micrographs of LR probe surfaces exposed to 20 µM 17 bp locking strand, added at t = 0, complementary to the hairpin sequence (**Table S1**) with Picasso localizations boxed in red at different time points. Scale bar = 3 µm. (**B**) Diagram of locking strand induced hairpin opening. (**C**) Localizations within FOV (1818 μm^2) over time for 4 surfaces (black dots) with the average (dark blue line) and standard deviations (light blue vertical lines). Note: FOV was adjusted to be a consistent, smaller area across all images to account for differences due to microscope drift.

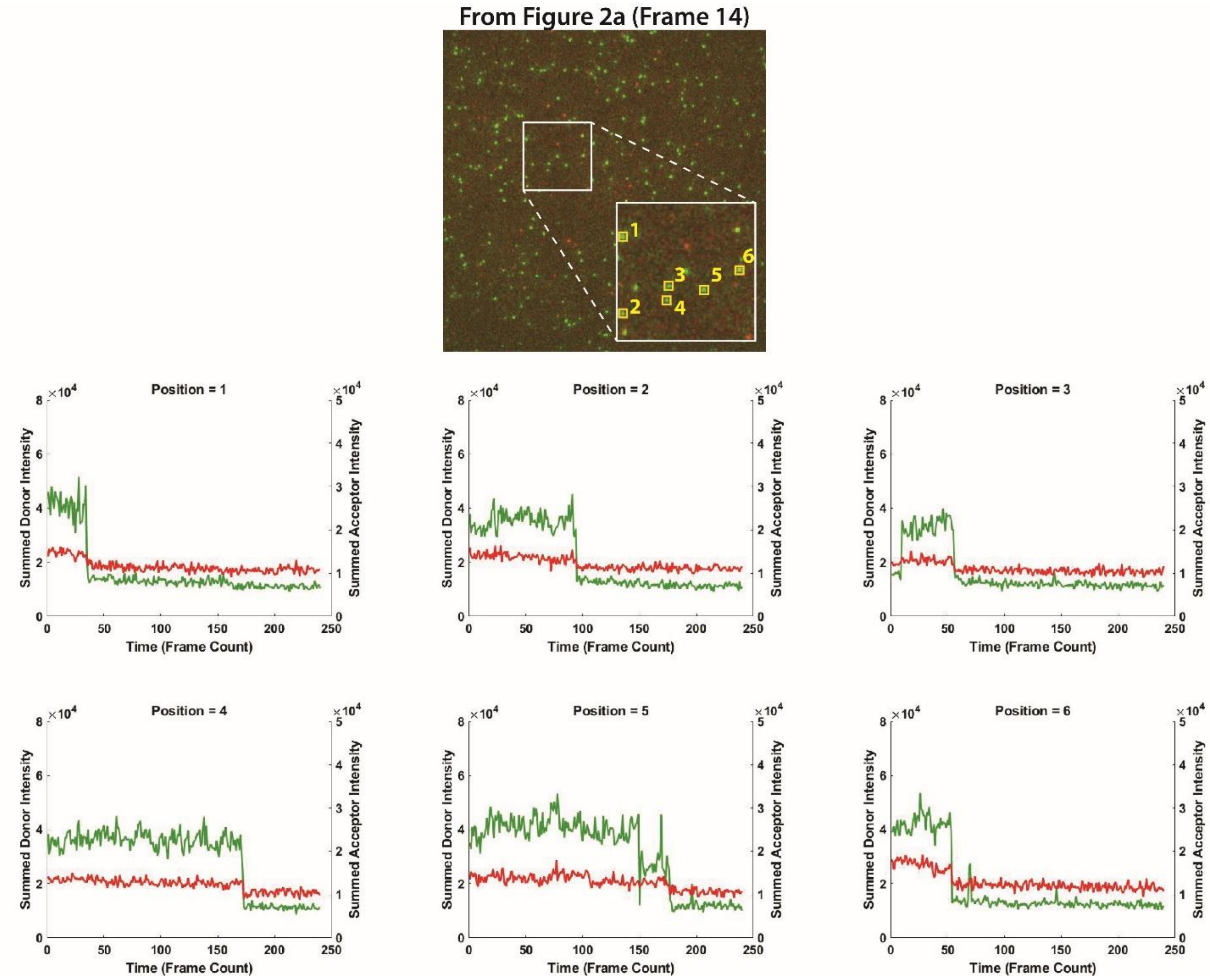

Figure S7. Additional traces from Figure 2a are plotted, showing single-step photobleaching for each of the molecules within this trace. This shows that each signal is from single molecules and that the surface does not induce clustering.

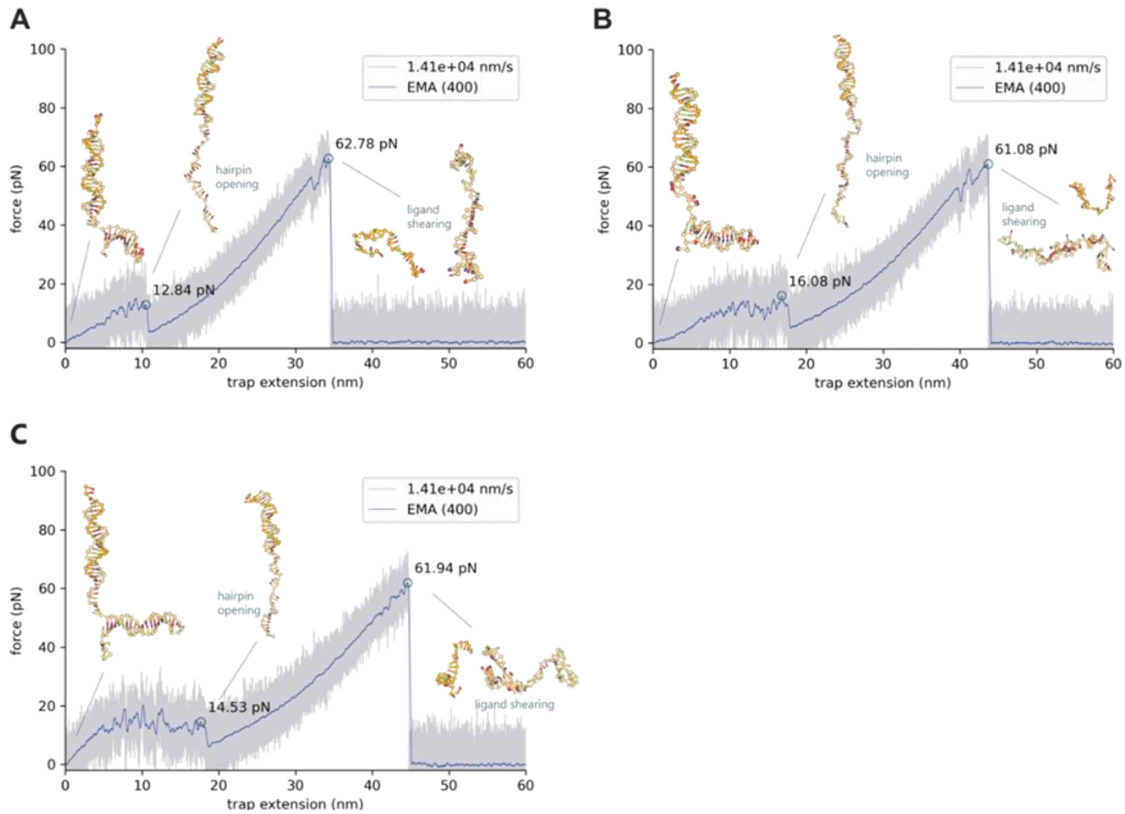

Figure S8. Force-extension curves of the LR probe as modeled by oxDNA. (A,B,C) Force-extension curves of LR probes with indicated force loading rates at each terminus. Gray: Raw data, Blue: Exponential moving average of 400 data points. Each graph represents the three separate simulation runs.

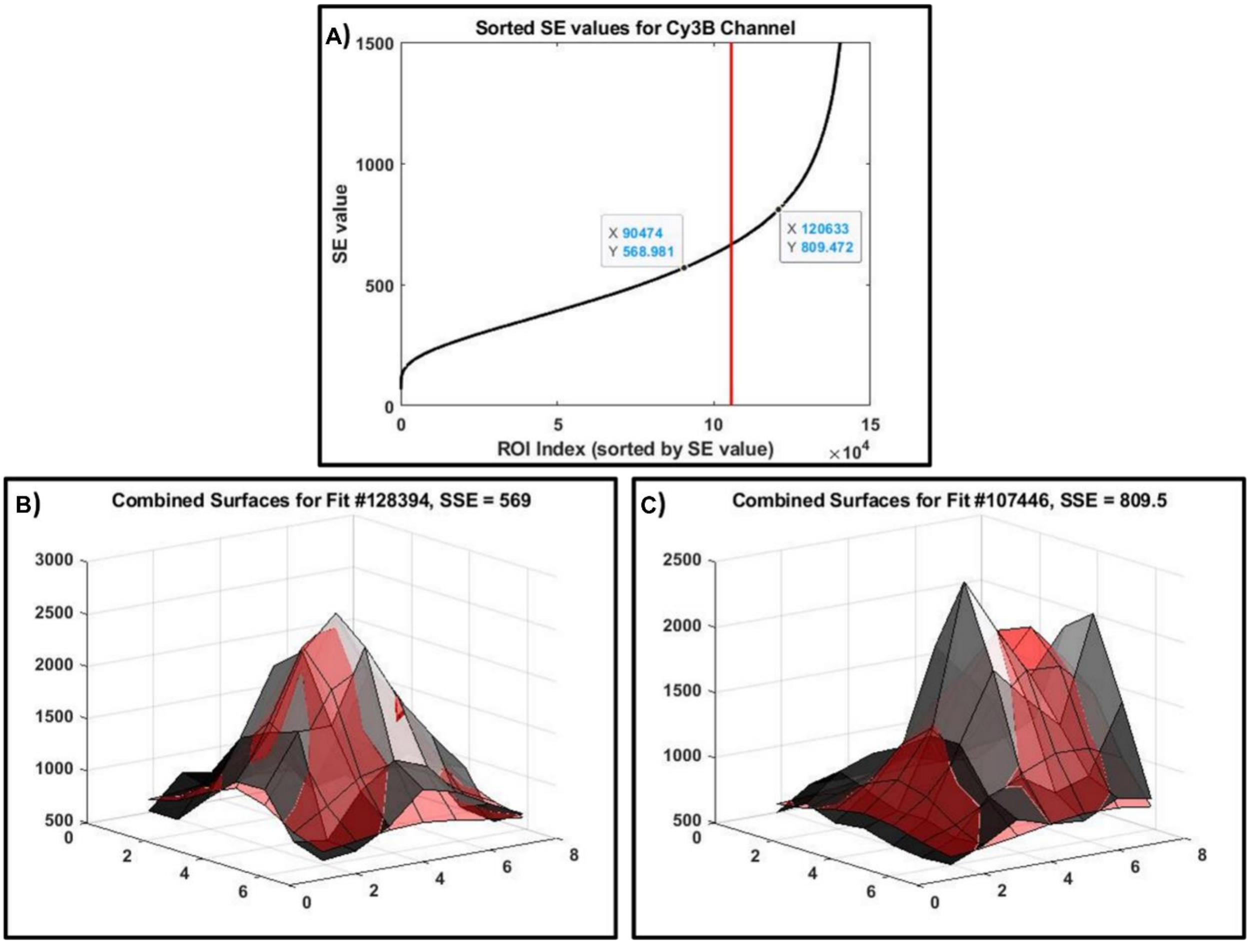

Figure S9. Identification of clean, single-molecule events from Gaussian fit data for further single-molecule analysis. (A) Sorted SE (fitting error) values for one measurement's worth of ROIs in the Cy3B channel. The vertical red line depicts the 70th percentile threshold (SE values below are accepted, SE values above are rejected). Two marked points have their surfaces and fits plotted below. (B) Surface and fit for an ROI matching the 60th percentile of SE values, this is representative of data which was used in further single-molecule analysis. (C) Surface and fit for an ROI matching the 80th percentile of SE values, this data was excluded from further single-molecule analysis.

SI Note 1 – LR Probe Concept

Our force loading rate (LR) probe is designed using two oligonucleotides that share a complementary domain (**Figure 1A, Table S1**). The top oligonucleotide (ligand strand) is chemically modified with a terminal cyclic peptide (cRGD) peptide to mediate integrin binding, while the bottom strand (anchor) covalently attaches the construct to the surface. The LR probe has three primary states which correspond to different force thresholds and can be identified by unique fluorescent signals: Closed, Open, and Sheared (**Figure 1C-E**). When the LR probe is at mechanical rest (F <4.7pN) the signal from both fluorophores is quenched by the corresponding BHQ2 dye (**Figure 1C** – closed). Upon integrin-ligand complex formation and a small (F >4.7 pN) force application, the hairpin domain is unfolded resulting in a signal from the Cy3B donor fluorophore (**Figure 1D** – opened). Once this force has increased to a high enough force threshold (F >47 pN) the ligand strand is sheared and the hairpin domain refolds producing a FRET signal from the donor-acceptor Cy3B-Atto647N pair (**Figure 1E** – sheared).

The key functional feature of this LR probe is that it generates an initial Cy3B turn-on signal at low magnitude of F >4.7 pN (opening event), and then this signal is dampened and followed by a FRET response when the force exceeds 47 pN (shearing event). Importantly, the appearance of both these signal events come from the same integrin-ligand interaction, and thus the force loading rate for each individual interaction can be inferred by taking the time difference between the two events. This process produces a sequence of fluorescence signals illustrated in the trace in **Figure 1F** which is used to calculate the force loading rate.

Table S1. Sequences of modified oligonucleotides as custom synthesized by Integrated DNA Technologies.

**Strand Name**	**Sequence(5' to 3')**
LR ligand	/5Hexynyl/TTT/iUniAmM/G CTG GGC TAC GTG GCG CTC TT/3AmMO/
LR anchor	/5Hexynyl/GT ATA AAT GTT TTT TTC ATT TAT ACT TTA AGA GCG CCA CGT AGC CCA GC/3AmMC6T/
Hairpin Locking Strand	GAA AAA AAC ATT TAT AC
cRGD-dsDNA-Biotin	/5AmMC6/TT TCG CAT CTG TGC GGT ATT TCA C/35OctdU/
dsDNA-Biotin compliment	GTG AAA TAC CGC ACA GAT GCG /3AmMC6T/

Experimental and Method Section

SI Note 2 – LR Probe Synthesis – Experimental Procedure

Cy3B-biotin-azide was synthesized as follows (**Figure S10**). To a solution of Biotin-(PEG_{11})-NH2 (1.0 mg – Broadpharm BP-21623) and iPr_2NEt (5.0 µL) in anhydrous DMSO (20 µL) was added Boc-Lys(N_3)-NHS (2 mg) in anhydrous DMSO (20 µL). After stirring for 60 min at room temperature, the reaction mixture was added to an 8:2 mixture of 18.2 MΩ MilliQ water and acetonitrile (200 µL) and filtered through a microcentrifuge filter (0.22 µm). The filtrate was subjected to reversed-phase HPLC (PLRP-S column, Agilent , 4.6 × 150 mm, 0.7 mL min−1 flow rate; solvent A: 0.05% TFA in 18.2 MΩ milli-Q water, solvent B: 0.05% TFA in acetonitrile; starting condition: 65% A + 35% B, 1% per min gradient B for 15 min) to obtain Biotin-PEG_{11}-Lys(N_3)-Boc as a colorless solid.

Biotin-PEG_{11}-Lys(N_3)-Boc was dissolved in trifluoroacetic acid (TFA, 20 µL) and dichloromethane (80 µL) and stirred for 1 hr at room temperature. The resulting mixture was diluted with 900 µL of acetonitrile, and the solution was concentrated to 50 µL. The mixture was added to 18.2 MΩ MilliQ water (200 µL) and filtered through a microcentrifuge filter (0.22 µm). The filtrate was subjected to reversed-phase HPLC (PLRP-S column, Agilent , 4.6 × 150 mm, 0.7 mL min−1 flow rate; solvent A: 0.05% TFA in 18.2 MΩ MilliQ water, solvent B: 0.05% TFA in acetonitrile; starting condition: 80% A + 20% B, 0.5% per min gradient B for 15 min) to obtain Biotin-PEG_{11}-Lys(N_3)-NH_2.

To a solution of Biotin-PEG_{11}-Lys(N_3)-NH_2 and iPr2NEt (1.0 µL) in anhydrous DMSO (10 µL) was added Cy3B-NHS (50 µg – GE Healthcare Life Science PA63101) in anhydrous DMSO (10 µL). After stirring for 30 min at room temperature, the resulting mixture was diluted with 18.2 MΩ MilliQ water (440 µL) and filtered through a microcentrifuge filter (0.22 µm). The filtrate was subjected to reversed-phase HPLC (PLRP-S column, Agilent, 4.6 × 150 mm, 0.7 mL min−1 flow rate; solvent A: 0.05% TFA in 18.2 MΩ MilliQ water, solvent B: 0.05% TFA in acetonitrile; starting condition: 80% A + 20% B, 1% per min gradient B for 21 min) to obtain Biotin-PEG_{11}-Lys(N_3)-Cy3B as a dark red solid.

To functionalize the LR anchor strand with A647N, A647N-NHS ester was dissolved in anhydrous DMSO and added to a solution of 0.5 mM LR anchor DNA, 0.1 M $NaHCO_3$, 1 X PBS for a final concentration of 10 % (v/v) DMSO, 3 mM A647N-NHS and left to react under sonication for 1 hour. The product was the separated by RP-HPLC (**Figure S11 A**) on an increasing linear gradient of 1% ACN/minute starting at 10%ACN using an analytical column specialized for oligonucleotides (Agilent 653950-702). The product was then reacted with PBS, 0.4 mM CuSO4, 2.0 mM THPTA, 2.0 mM sodium ascorbate, 2.5 mM Cy3B-biotin-Azide at 37° C for 2 hrs in a copper assisted cycloaddition reaction. The product was then separated on a linear gradient of 0.5% B(ACN) increasing per minute starting 22.5% B, solvent A is 0.1M TEAA MQ-H_2O (**Figure S11 B**). The product was then further separated on a different column (Agilent PLRP-S) at 60° C (**Figure S11 C**).

To functionalize the LR ligand and dsDNA-biotin strands with the ECM mimetic peptide Cyclo[Arg-Gly-Asp-D-Phe-Lys](cRGD), 100 nmol of the peptide (Fisher Scientific 50-168-6290) was reacted with 200nmol NHS-Azide (ThermoScientific 88902) in 10 µL anhydrous DMSO for 6 hr. at room temperature(**Figure S12 A**). The product was separated by RP-HPLC with a flow rate 1mL/min; solvent A: MQ-H2O 0.05% TFA, solvent B: ACN, 0.05% TFA, with the initial condition 10% B with a linear gradient of increasing 0.5% B per min (**Figure S12 B**). For this product, mass spectrometry (**Figure S12 C**) was carried out on an Applied Biosystems 4700 Proteomics Analyzer Matrix-assisted laser desorption/ionization-time of flight (MALDI-TOF) mass spectrometer by dissolving the product in saturated solution of α-cyano-4-hydroxycinnamic acid in 49.4% (v/v) H2O, 49.4% acetonitrile, 0.1% TFA and allowing to dry.

The LR ligand strand was reacted in a copper assisted cycloaddition reaction with 2.5 mM cRGD-azide in PBS, 0.4 mM CuSO4, 2.0 mM THPTA, 2.0 mM sodium ascorbate at 37° C for 2 hrs. The product (product 3) was separated on RP-HPLC with an increasing linear gradient of 1% ACN/min

starting at 10%ACN (**Figure S13 A**). Product 5 was then reacted with 3 mM BHQ2-NHS (Biosearch Technologies BHQ-2000S) in PBS, 70% (v/v) DMSO, and 0.1 M $NaHCO_3$. The final LR ligand product(product 4) was then separated on RP-HPLC with a gradient of 1%ACN/min. starting at 25% ACN (**Figure S13 B**).

To synthesize functionalize cRGD-dsDNA-Biotin strand with cRGD and biotin, the strand was reacted with cRGD-azide in PBS, 0.4 mM CuSO4, 2.0 mM THPTA, 2.0 mM sodium ascorbate at 37° C for 2 hrs and separated on RP-HPLC with an increasing linear gradient of 1% ACN/minute starting at 10%ACN (**Figure S14 A**). The product was then reacted with PC biotin-PEG4 NHS (Click Chemistry Tools 1225-1)(note: the photocleavable functionality of this product was not employed in this study) in PBS, 50% (v/v) DMSO, 0.1 M $NaHCO_3$ for 2 hrs and HPLC purified on a stepped gradient starting at 10% ACN and increasing at 1.5 %ACN/min. for 10 min. followed by 0.5%ACN/min. for 30 min. (**Figure S14 B**). Both LR Ligand and cRGD-dsDNA-Biotin were HPLC purified using an analytical column specialized for oligonucleotides (Agilent 653950-702).

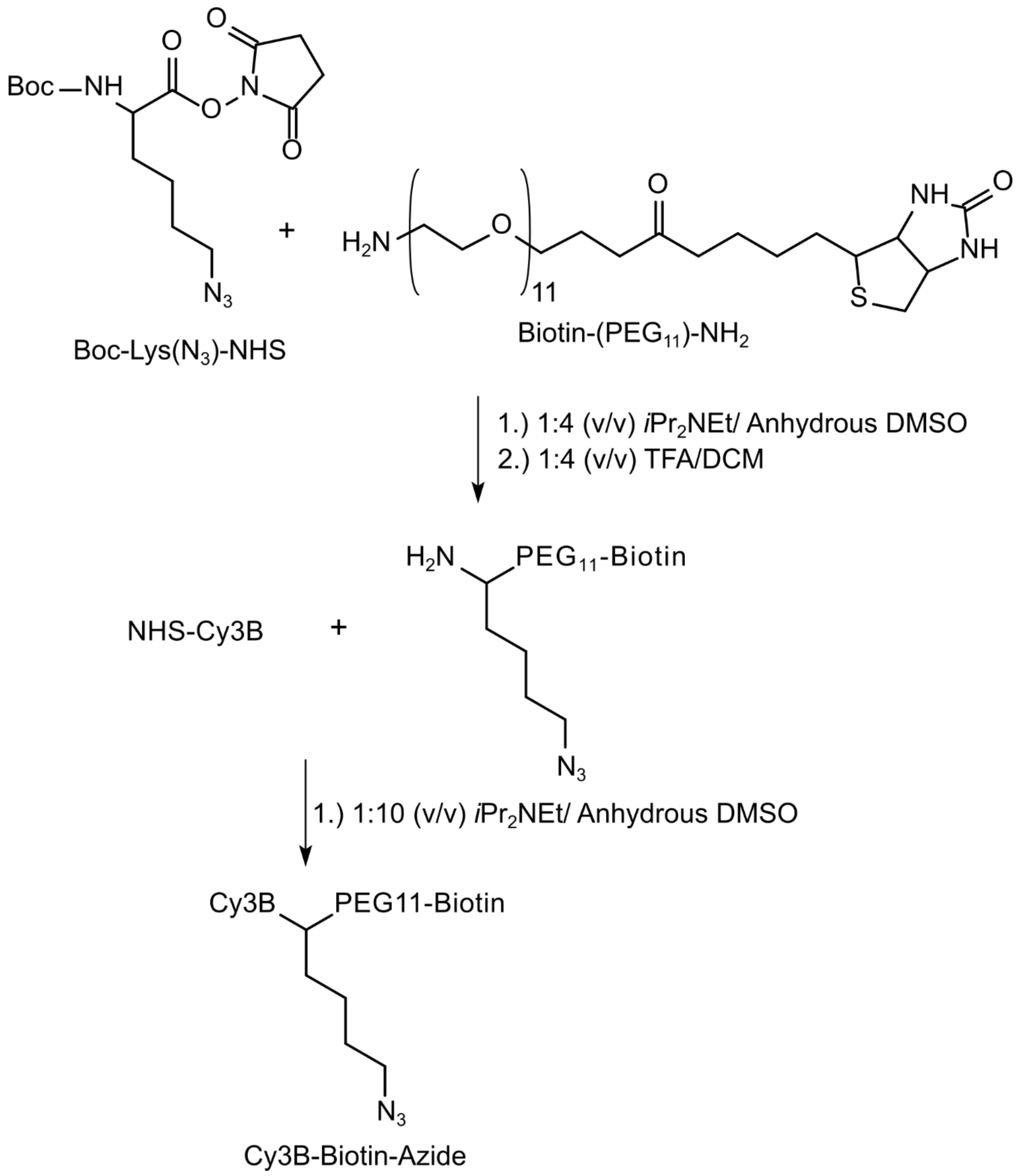

Figure S10. Synthesis of Cy3B-Biotin-Azide. Products were purified by RP-HPLC after each step. Specific details for all reaction conditions can be found in **SI Note 2** above.

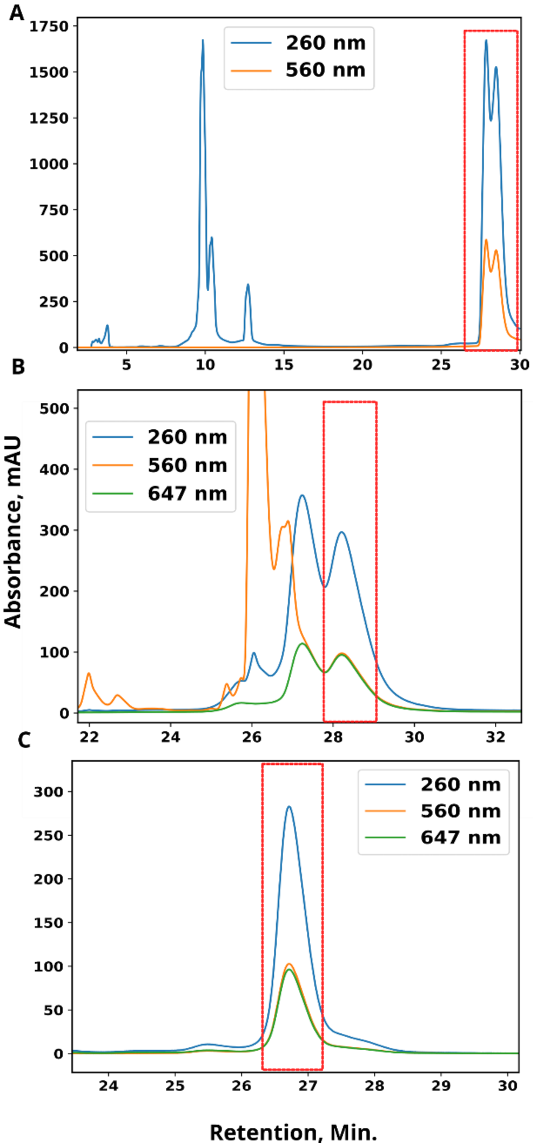

Figure S11. LR anchor HPLC chromatograms. (A) Product collected from reaction with LR anchor with A647N-NHS (product 1). (B) Product collection from reaction with product 1 with Cy3B-biotin-azide purified (product 2). (C) Final LR anchor product collection from re-purification of product 2 at 60° C. Collected fractions boxed in red.

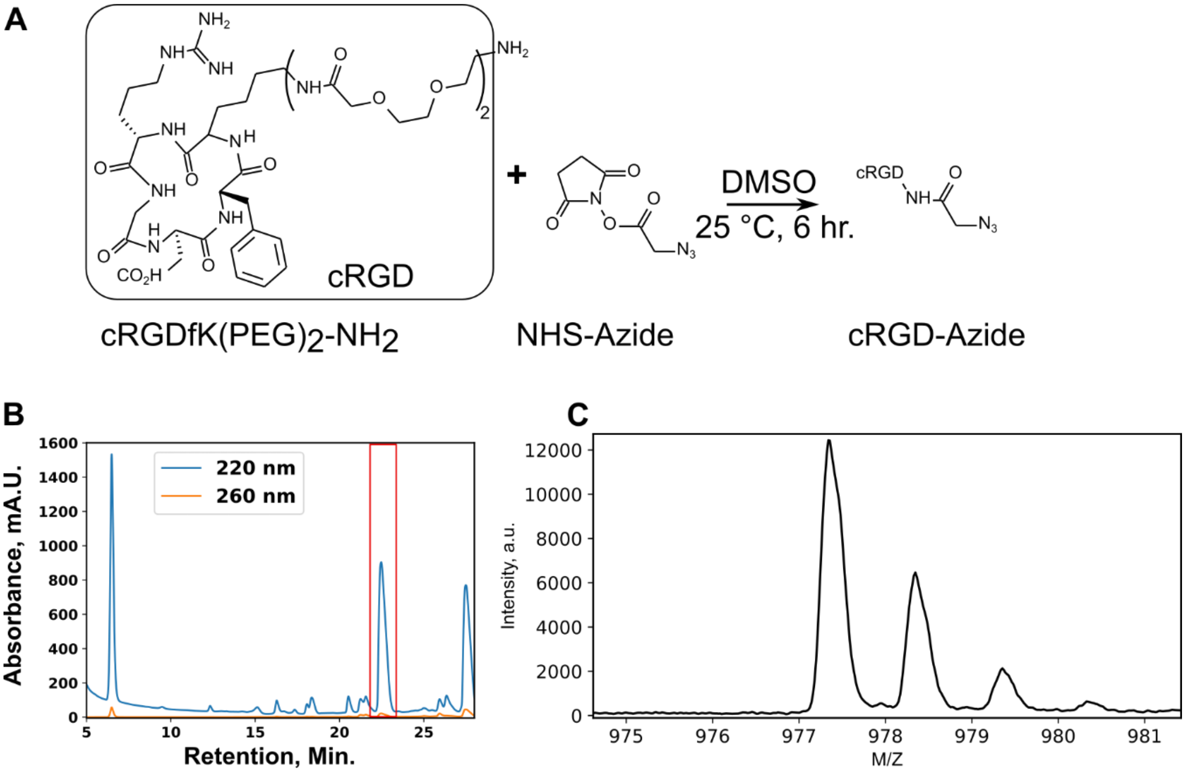

Figure S12. Synthesis and purification of cRGD-Azide. (A) Chemical structures and reaction between cRGDfK(PEG)2-NH2 and NHS-Azide. (B) HPLC chromatogram of cRGD-Azide with collected fraction boxed in red. (C) MALDI-TOF mass spectrometry of cRGD-Azide with calculated mass: 977.03, measured mass: 977.02. Collected fraction boxed in red.

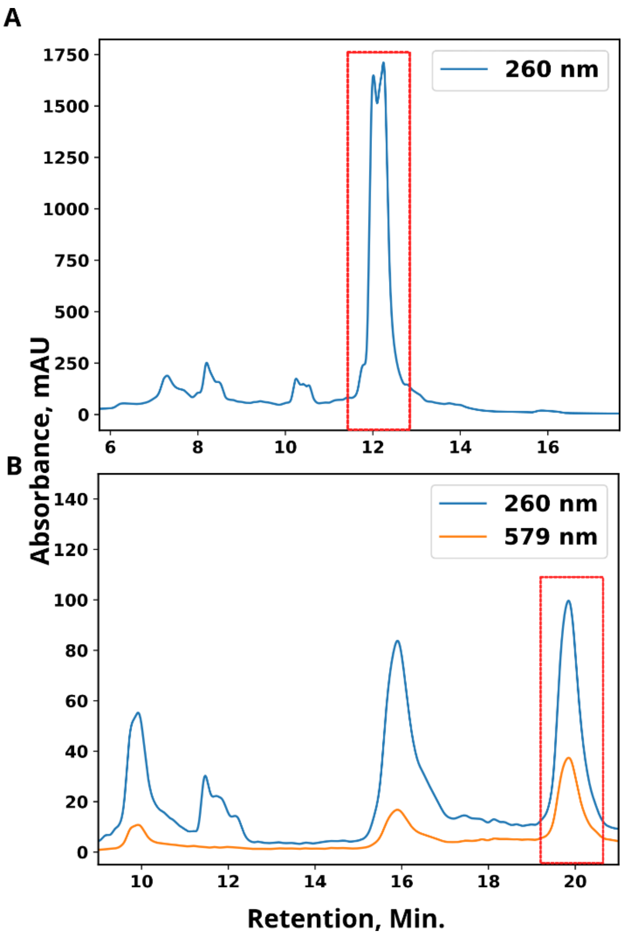

Figure S13. LR ligand HPLC chromatograms. (A) Product collected from reaction with cRGD-N3 with LR ligand strand (product 3). (B) Final LR ligand product collected from the reaction of product 3 with BHQ2-NHS (product 4). Collected fractions boxed in red.

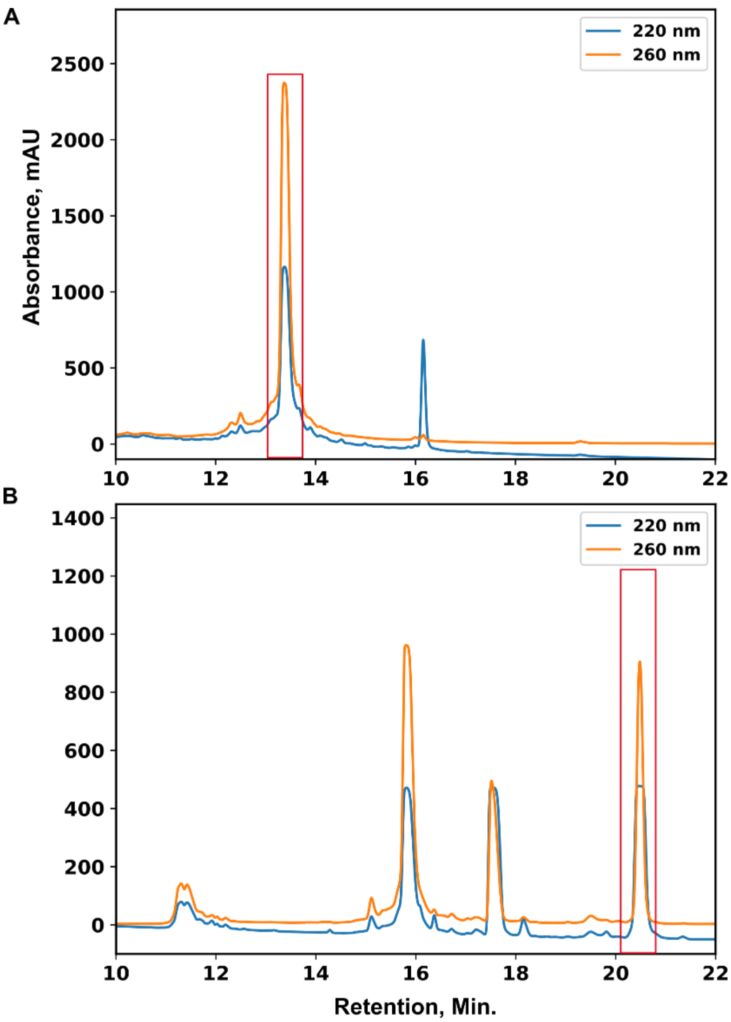

Figure S14. cRGD-dsDNA-biotin HPLC chromatograms. (A) Product collected from reaction with dsDNA-Biotin DNA strand with cRGD-Azide(product 1). (B) Product collection from reaction with product 1 with PC biotin -PEG4 NHS. Collected fractions boxed in red.

SI Note 3 – Modeling of F_{shear} with variable loading rate

It is important to note that the force at which a DNA duplex shears (F_{shear}) is dependent on the loading rate of the force applied. As our observed loading rate is dependent on this value, we sought a model for F_{shear} which takes into account loading rates which are comparable to our empirically determined values (~1 pN/sec). Recent work by Liu and Yan modeled a loading-rate-dependent value for the rupture force, and experimentally validated this model on several strands[21]. In their work, they also included a user-friendly code which allows readers to model a strand of interest at varying temperatures and loading rates. We utilized this code to model the F_{shear} of our shearing strand at 25C under a loading rate of 1 pN/sec, yielding a predicted F_{shear} = 47 pN which takes into account our loading rate. The code which implements their model is made available and can be found here (https://colab.research.google.com/drive/1i5FdSSwPi-jJMEtC7aszWtu_vC4k2AlG?usp=sharing), and the results which model the LR probe are reproduced in **Figure S15** below.

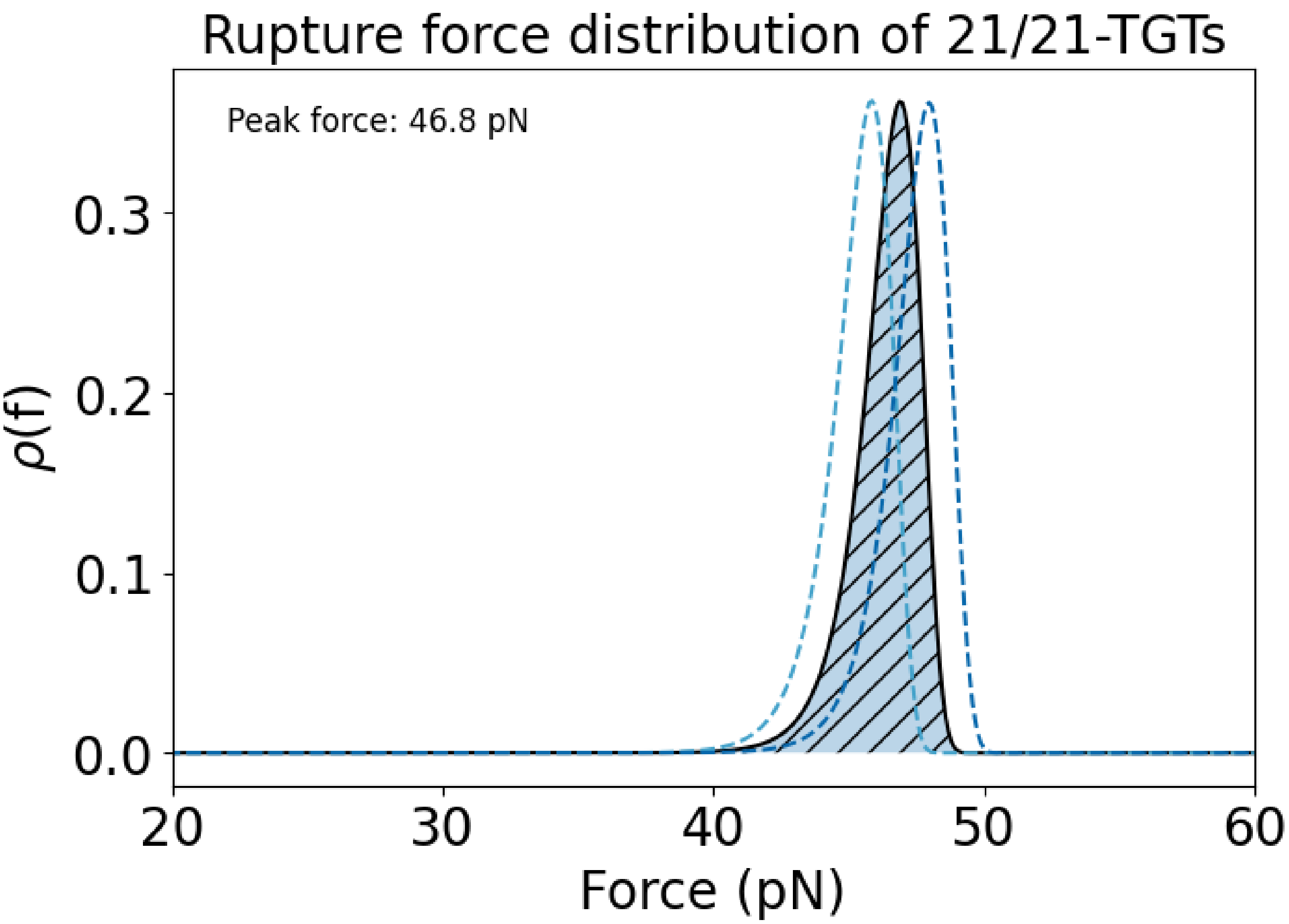

Figure S15. Results from a loading-rate dependent shearing model. Input temperature for the model is 25°C and input loading rate is 1 pN/sec.

SI Note 4 – LR Probe Surface Preparation – Experimental Procedure

Square 25 mm glass coverslips (Fisher Premium Cover Glass 12-548-C) (**Figure S2**) were incubated in a piranha solution (3:1 v/v concentrated sulfuric acid/30% by volume hydrogen peroxide) for 30 min on a fluorocarbon surface rack. Then coverslips were washed in MQ-H_2O by transferring the rack serially from beaker to beaker in six separate beakers. The same procedure was carried out to wash the coverslips in pure ethanol three times using separate beakers. The coverslips were then transferred to a beaker with 3%(v/v) (3-aminopropyl)triethoxysilane(APTES) (Sigma 440140) in ethanol, covered with parafilm and gently shaken on a rotary shaker for 45 min. The aminated surfaces were then washed three times in ethanol and baked at 100 °C for 20 min. The surfaces were then placed in a sandwich arrangement with 150 uL solution 0.5 M K_2SO_4, 0.1 M $NaHCO_3$, 30 mg/ml Biotin-PEG(5kDa)-NHS(Biopharma PEG HE0412024-5K) between two surfaces for 45 min. Each surface was then washed three times by transferring 1 mL MQ-H_2O on the surfaces with excess wash solution wicked away from the surfaces with a Kim wipe after upon the final wash to remove as much MQ-H_2O as possible. This process was repeated two more times for a total of 3 Biotin-PEG(5kDa)-NHS reagent exposure sandwiches and washings. Surfaces were then sandwiched with 150 uL 50 mg/ml mPEG(2kDa)-NHS(Biopharma PEG MF001024-2K), 1 mg/ml NHS-acetate(ThermoScientific 26777) between two surfaces and incubated overnight for at least six hours. These PEG surfaces are commonly used in single-molecule experiments and exhibit minimal non-specific binding, as noted by the absence of LR-probe clusters seen in **Figure S7**.

Before mounting, surfaces were washed as above three times with 3x MQ-H_2O, then three times with pure ethanol, then dried with compressed air passed through a 0.22 μm mesh syringe filter. Surfaces were then mounted to the 384 well plate sticky bottom (Grace Bio-Labs 206384) taking care to ensure the biotinylated side faces the interior of the well plate well.

On the day of imaging, the well (150 μL volume) to be used was washed with 1 mL of pure ethanol by pipetting the ethanol directly into the well while simultaneously aspirating solution out from the top edge

of the well. The well was then incubated with pure MQ-H_2O for 2-5 min, then washed with 1 mL MQ-H_2O, then wash with 200 µL phosphate buffered saline solution (PBS) pH 7.4. To functionalize the surfaces with streptavidin, 6 µL 0.1mg/ml streptavidin (Rockland-Inc. S000–01) in PBS was added to the well and incubated for 30 minutes. The well was then washed with 200 µL PBS and exposed to biotinylated LR probe. Annealed LR probe was diluted from the concentration of 1.0 µM to 400 pM, 0.75-1 µL of which was added to the surface pipetting up and down in the well solution 4-7 times and incubated for 45 min. The interaction between streptavidin-functionalized surface and biotinylated LR probe is what functionalizes the surface with the probe. To add non-fluorescent supplemental ligand to promote cell adhesion 1 µL 1.0 µM biotinylated dsDNA-cRGD ligand (**Table S1**) was added to the well without prior washing by pipetting up and down 4-7 times as previously and incubated for 45 min. Surfaces were then finally washed with 200 µL PBS.

SI Note 5 – Live Cell Imaging – Experimental Procedure

Cells were detached from plates 0.25% (wt/vol) trypsin, 2.21 mM EDTA (corning) and resuspended in normal culture media at a 1:20 v/v ratio, centrifuged at 250 X G for 4 minutes and resuspended in Fluorobrite DMEM (Gibco A1896701) supplemented with 5% serum imaging media. The imaging well was washed with 200 µL imaging media before adding 400-800 cells to the 384 well plate well (area density of 0.32-0.64 cells/cm2). Cells were then incubated for 25 min. at 37 °C 5% CO2 for 25 minutes before imaging.

Cells were then imaged at room temperature on a Nikon Eclipse Ti microscope driven by the NIS Elements Advanced Research software. The microscope objective used was CFI Apo 100X (numerical aperture 1.49) objective (Nikon). The optical system includes a total internal reflectance fluorescence (TIRF) variable mirror launcher and a Nikon Perfect Focus System, an interferometry- based device which corrects z-drift of the stage. Reflection interference contrast microscopy (RICM) images were captured using a filter cube (Chroma 97270 SRIC C168785) and by a Lumen Dynamics X-Cite 120 LED light source. Fluorescence excitation of samples through the optical system was carried out, 561nm (50 mW, used at 0.5% LP), and 640 nm (100mW, used at 1% LP) lasers. A Chroma Quad (Chroma TRF89901 ZT405/488/561/640rpc) TIRF filter cube was used in the imaging acquisition of all three fluorescence channels. The optical configuration included the Andor TuCAM dual camera system comprised of two Andor DU-897 X-9319 cameras with a conversion gain of 3 and gain multiplier of 800 imaging at 50-100 ms exposure times. A Chroma ZT647rdc dichroic mirror was placed in the optical path to deflect light of wavelength < 640 nm, comprising the emission of Cy3B, to the second camera **(Figure S1**).

To correct for the offset between image planes of the two cameras, slides with Tetraspec Beads (Invitrogen T7281) fluorescent in both channels were imaged and planes were aligned using an affine transform based algorithm on the imageJ software[19]. Timelapse Images were drift corrected using an

imageJ plugin based on software written by Preibisch *et. al.*[19]. Cells were imaged at 10, 20, and 60 second intervals between fluorescence acquisitions taking two frames of three fluorescence images at each acquisition interval: A647N, then Cy3B and Cy3B sensitized FRET to A647N simultaneously on both cameras. To ensure that the timepoint of the opening and shearing events are not due to stochastic intensity fluctuation two steps were taken. The first is that we employed a dual camera system which splits the emission of Cy3B and A647N to two separate cameras which record Cy3B and fluorescence and FRET micrographs simultaneously (**Figure S1**). This ensures that the Cy3B and FRET channel intensities are synchronized in time. The second is that for every frame which is separated in time by the frame interval, two Cy3B and FRET fluorescence micrographs were taken separated by a 100 ms exposure time delay and averaged to produce a micrograph with an average intensity between the first and second micrograph at the specific frame. We applied this measurement to cells spreading on LR probes at 10, 20, and 60 sec. frame intervals and found that 10 sec. was sufficient to capture Cy3B opening traces that lasted many frames. (**Figure S16-S19**).

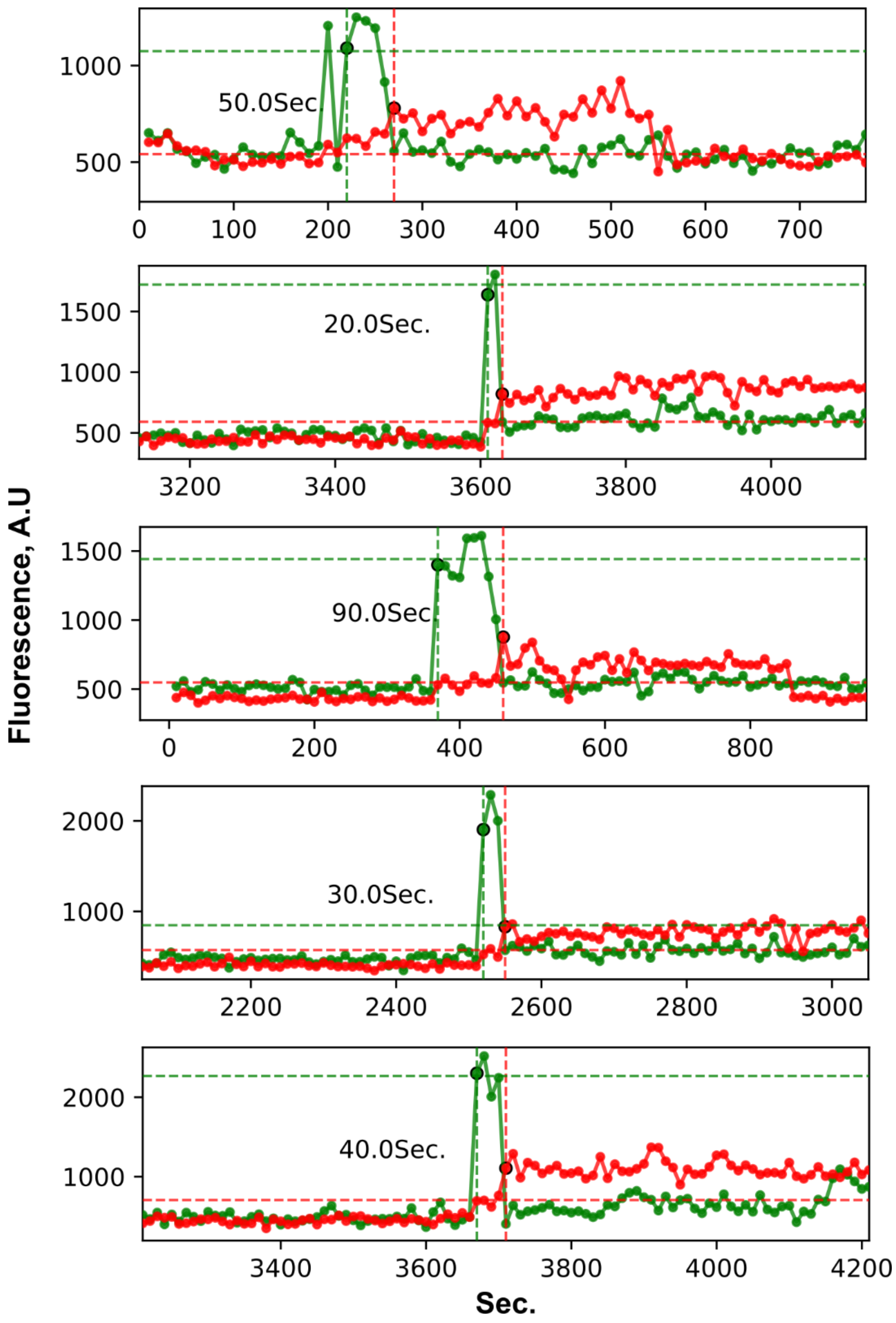

Figure S16. Representative loading rate trace localizations at a 10 sec. frame interval.

Loading rate trace localizations with the time between shearing and opening demarcated as calculated from the difference between the instance of Cy3B(green trace) turn on (green dashed vertical line) and the instance of simultaneous Cy3B decrease followed by FRET channel(red trace) increase(red dashed vertical line). Cy3B average intensity during turn on demarcated by vertical dashed line, and average intensity during FRET demarcated by red dashed line.

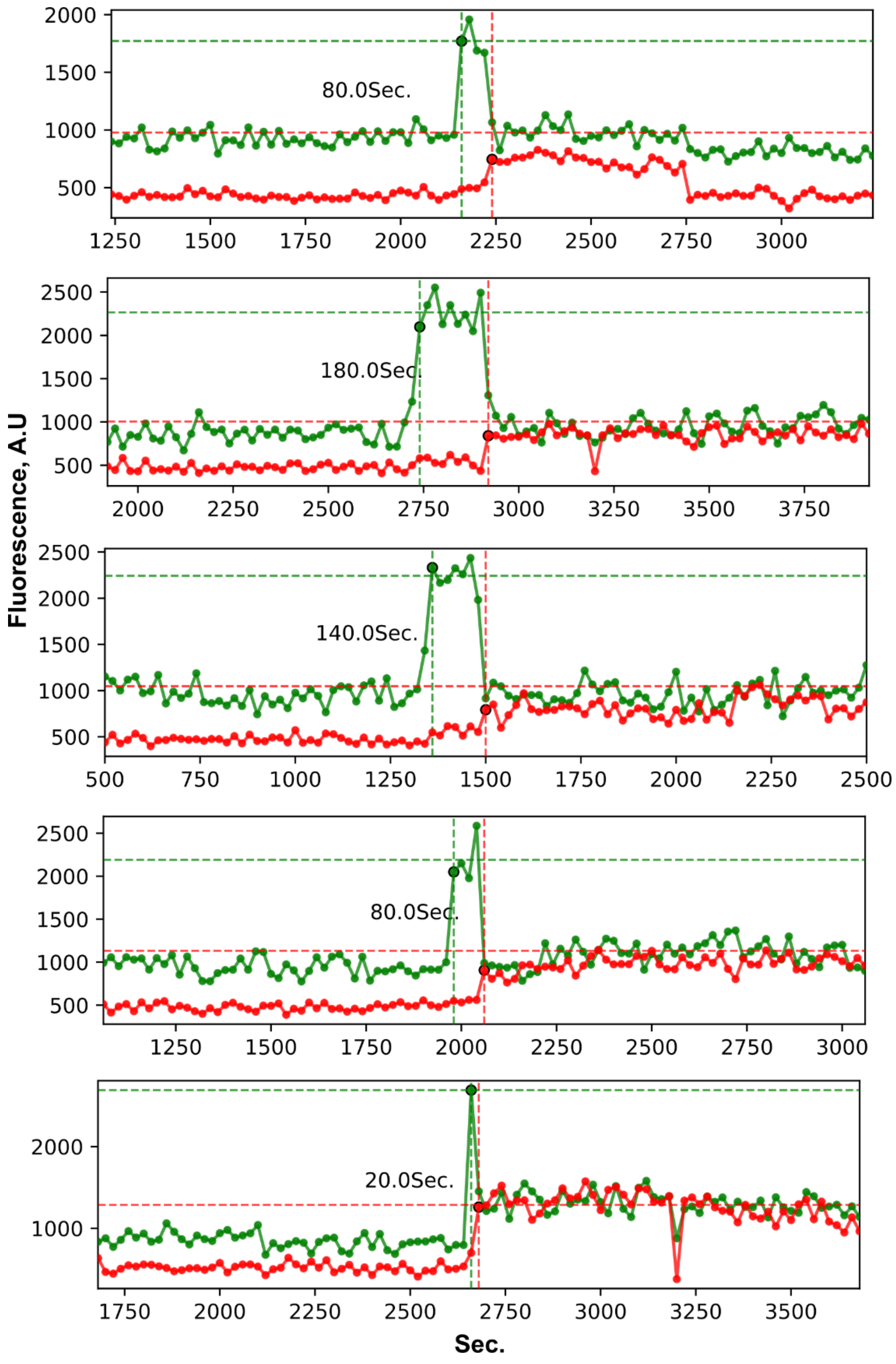

Figure S17. Representative loading rate trace localizations at a 20 sec. frame interval. Loading rate trace localizations with the time between shearing and opening demarcated as calculated from the difference between the instance of Cy3B(green trace) turn on (green dashed vertical line) and the instance of simultaneous Cy3B decrease followed by FRET channel(red trace) increase(red dashed vertical line). Cy3B average intensity during turn on demarcated by vertical dashed line, and average intensity during FRET demarcated by red dashed line.

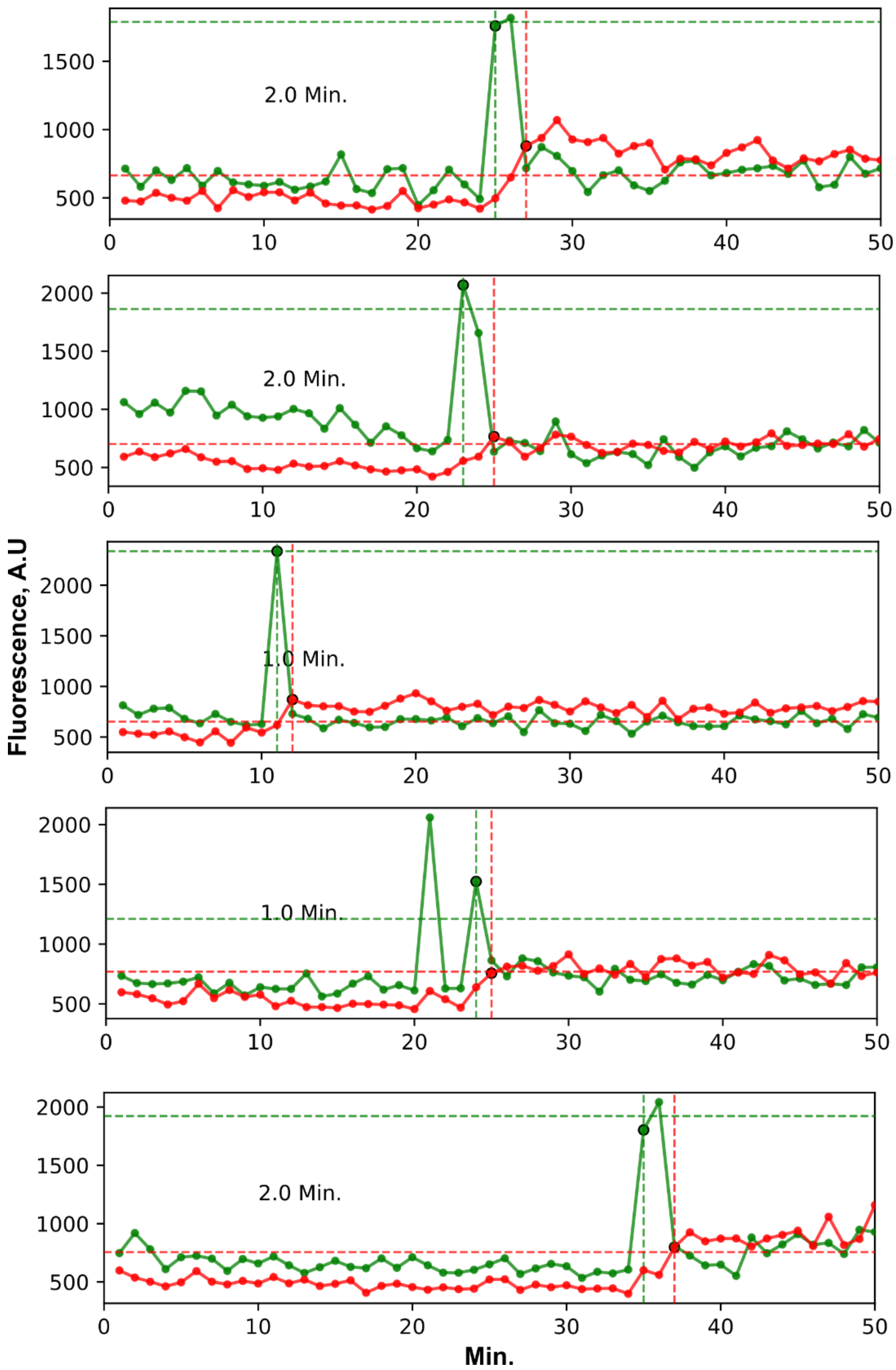

Figure S18. Representative loading rate trace localization at a 1 min. frame interval. Loading rate trace localizations with the time between shearing and opening demarcated as calculated from the difference between the instance of Cy3B(green trace) turn on (green dashed vertical line) and the instance of simultaneous Cy3B decrease followed by FRET channel(red trace) increase(red dashed vertical line). Cy3B average intensity during turn on demarcated by vertical dashed line, and average intensity during FRET demarcated by red dashed line.

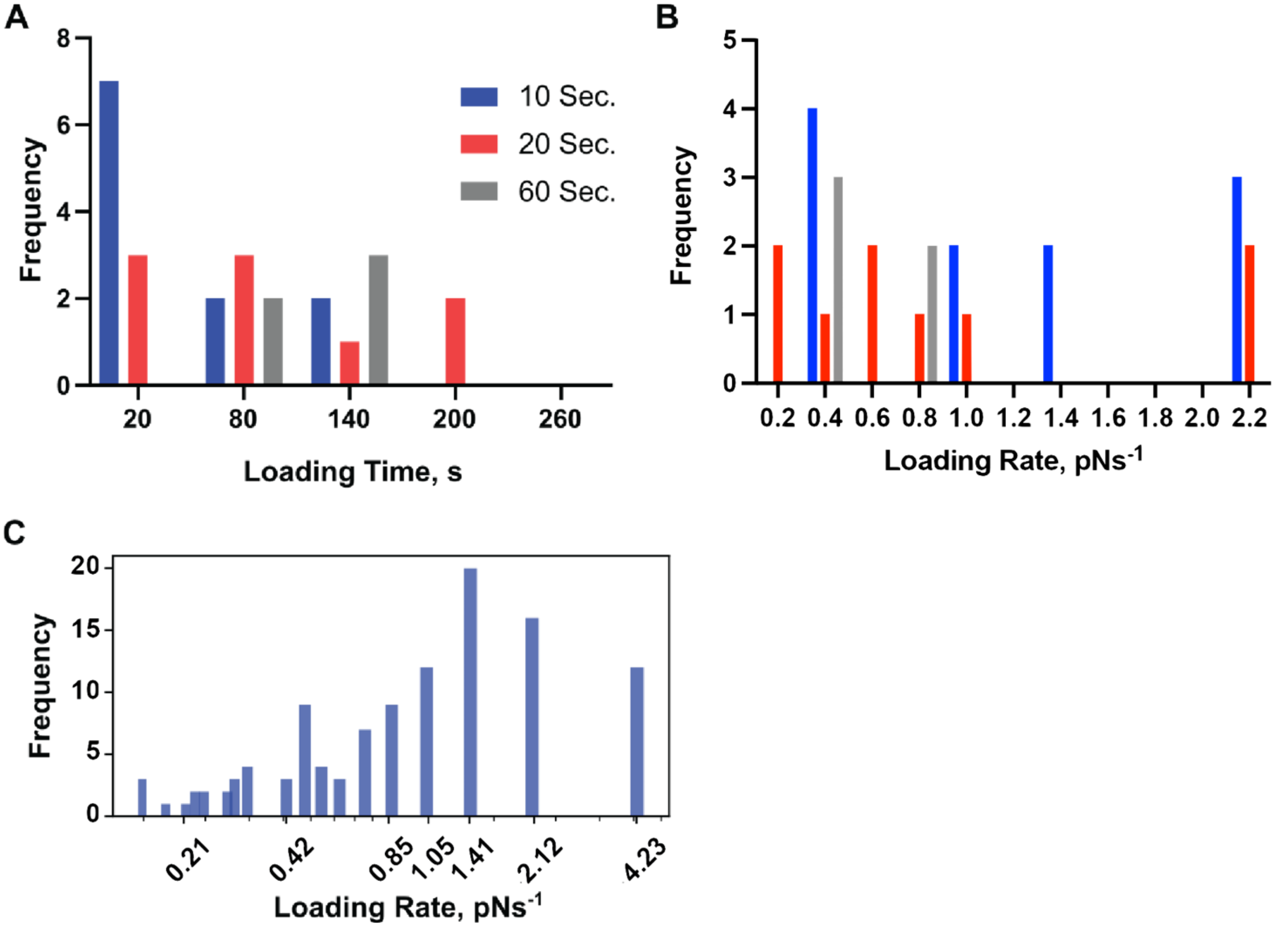

Figure S19. Manually identified loading time and linear loading rate for different frame intervals. (A) Loading time as derived from opening-shearing traces for cells from 10, 20 and 60 sec, N =1 cell for each frame interval. frame intervals. (B) Loading rates assuming a linear force ramp between the opening and shearing signals and that opening occurs at 4.7 pN and shearing occurs at 47 pN. (C) Manually identified loading rate for cells collected at a 10 sec frame interval, N = 8 cells.

SI Note 6 – OxDNA Modeling of the LR Probe

In addition to using DNA secondary structures and sequences with previously studied force induced transitions[1,12], we modeled the opening force and the ligand shearing force of our novel construct using the oxDNA2 model[13]. MD simulations were run on the annealed DNA hairpin strands (**Figure S8**) at an extension rate of 1.41 x10^4 nms^{-1} along the z-axis.

Temperature and [Na+] were set to 37°C and 0.156 M respectively to mimic in vitro experimental conditions and the following parameters were used in oxDNA(**Table S2**). The terminal nucleotides with the ligands and anchor were added with harmonic traps each with stiffness constants k_1 and k_2 (equivalent to springs in oxDNA) of 11.40 pNnm^{-1}. The combined effective trap stiffness k_{eff} can be calculated using **Equation S3,** where k_1 and k_2 are the stiffness constants of the two traps between which the LR probe construct is pulled.

Table S2. Settings used for oxDNA modeling of LR probe force transitions.

Steps	5e9
diff_coeff	2.5
thermostat	john
T	37C
interaction_type	DNA2
Newtonian_steps	103
use_ave_seq	1
verlet_skin	0.05
salt_concentration	0.156
dt	0.005

Equation S3. Combined trap stiffness

$$\frac{1}{k_{eff}} = \frac{1}{k_1} + \frac{1}{k_2}$$

An extension rate of 1.4×10^4 nm/s along the z-direction was used to move one of the traps on the nucleotide to which the cell adhesion ligand is attached. To obtain the force-extension curve in each simulation, we extracted the extensions of harmonic trap from the attached nucleotides and then projected it along the z-axis. The force is calculated by multiplying the total projected extension with k*eff*. The data

points were then smoothed with a 400-point exponential moving average (EMA) of the data points using python (10.1038/s41586-020-2649-2). The rupture force was estimated by picking the peak at the point of rupture using SciPy find_peaks module (10.1038/s41592-019-0686-2). From the simulations, the opening of hairpins remained within the range of 12-16 pN (**Figure S8**). The difference in hairpin opening force with regards to the experimental estimates can be reasoned using the following factors. 1) Experiments use a constant force clamp to estimate the 50% probability of hairpin dissociating at a particular force within a defined interval of time (e.g., 2 sec) whereas in simulation the probes are far from equilibrium. This leads to higher force estimation for hairpin opening opening[14,15]. The constant force is incrementally increased in experiments, while the simulation experiments were performed by applying a defined velocity to the nucleotide. In a typical force ramp setup, the rupture force of a DNA duplex is highly dependent on the loading rate (i.e., the higher the loading rate the higher the rupture force). 2) The loading rates used in simulation are much faster compared to experiments due to computational resource limitations.[16,17] 3) oxDNA simulations have an inherent level of stochasticity that results in small variations in the resulting force of mechanical transitions(**Figure S8 A-C**). These simulations yield a force difference between the unfolding and shearing events of 47.69 ± 1.73 pN, near to the 42.3 pN force difference inferred from the previously reported mechanical transitions of 4.7 and 47 pN for the opening and shearing domains respectively.

SI Note 7 – Automated Analysis of Single-Molecule Data

Custom Matlab codes were written and implemented on each measurement for automated calculation of the single-molecule loading rate. Input parameters for the codes were first optimized on a representative dataset and then applied uniformly throughout all datasets during analysis. The custom code was written to perform 4 primary steps:

1) Finding optimal single-molecule events using Gaussian fitting
2) Obtaining time-traces of single-molecule events
3) Identifying key timepoints within time-traces and sorting events
4) Compiling kinetics of LR probe-rupturing event

The analysis used to perform these four steps and the logic behind them are described in detail below:

Note: For the following SI sections each step of the analysis will be performed on a single representative data set for the sake of simplification. The shown data analysis process is repeated in an identical manner across all data within the experiment.

7.1 Analysis of SM Data 1: Finding optimal single-molecule events using Gaussian fitting

The first step is to identify single molecules which can be analyzed independently from all other molecules. Fluorescent spots were identified by scanning the image for localizations which satisfied three criteria: 1) Intensity above a certain threshold (1.85 or 2.4 times the value of the background for the FRET and Cy3B channels, respectively), 2) local maxima in the x and y-direction, and 3) a peak prominence above 20 counts (to minimize the effects of shot noise). Pixels which satisfied these criteria were marked as a region of interest (ROI), and a 7x7 region centered on the localization was fit to determine the quality of that ROI. In general, a diffraction limited spot made from a fluorescent single molecule should be well fit with a 2D-Gaussian fit with the form (**Equation S1**):

Equation S1. 2D-Gaussian fit for a diffraction limited spot.

$$\boldsymbol{Fit(x,y)} = \boldsymbol{I} * \exp\left(-\left(\frac{(x-\boldsymbol{x_0})^2}{2(\boldsymbol{\sigma_x})^2} + \frac{(y-\boldsymbol{y_0})^2}{2(\boldsymbol{\sigma_y})^2}\right)\right) + \boldsymbol{b}$$

Where x_0 and y_0 are the Gaussian's center in x and y, respectively, σ_x and σ_y are the Gaussian spread in x and y, I is a normalization factor, and b is the background. In the above equation the variables of the fit are marked in red and adjusting these variables changes the output 2D Gaussian surface, F(x,y), which is then compared to the raw data in order to quantitatively determine a "best fit" (typically a local maximum within this parameter space). A representative ROI showing such a fit and how the individual parameters affect the fit is displayed below in **Figure S20**:

The "best fit" was quantitatively determined using a form of Maximum Likelihood Estimation (MLE) which was chosen based off of the assumption that noise in the measurement follows a Poisson Distribution[10], and follows the form:

Equation S2. Summed error from the Poisson distribution of the data and fit.

$$\mathbf{SE} = \sum\left(\boldsymbol{Data(x,y)} * \ln\frac{\boldsymbol{Data(x,y)}}{\boldsymbol{Fit(x,y)}}\right)$$

Where SE if the summed error (inverse fit quality), ln is the natural logarithm, Data(x,y) is the raw data, and Fit(x,y) is the fitted surface from **Equation S1**. The error is calculated at each pixel within the ROI (here a 7x7, so 47 total points) and those errors are summed to give FQ. Intuitively, the natural logarithm of the ratio Data(x,y)/Fit(x,y) changes sign depending on which is larger, and so Fit(x,y) is normalized to the total intensity of Data(x,y) to avoid fit convergence on extremely large/small values. Lastly, custom bounds were added to the definition of FQ to restrain its parameter space to p hysically meaningful values given the single-molecule nature of the measurement (e.g. similar σ_x and σ_y values, peaks within 1.5 pixels of the ROI center, S:N ratio > 1, etc.).

In a given measurement there are often upwards of a hundred thousand ROIs. Once each ROI is fit and assigned a summed error, the top 70th percentile of SSEs are selected for further analysis. **Figure S9** shows all SE values in the Cy3B channel for a representative experiment, along with the 70th percentile threshold and representative fits which were slightly better and slightly worse than the threshold cutoff. As can be seen from **Figure S9 B**, fits below the 70th percentile of SE values fit well to the 2D Gaussian model for an expected single-molecule measurement. In contrast, the primary contributor to high SE values is

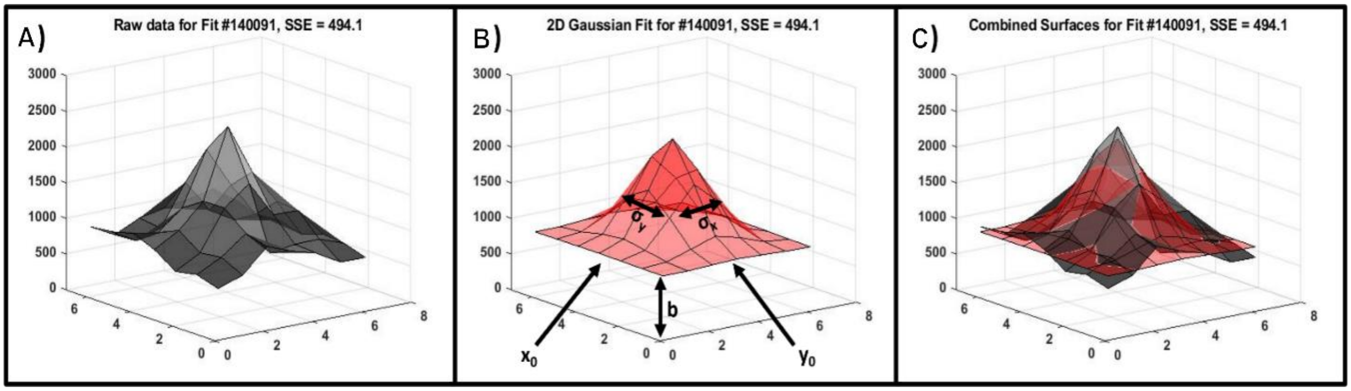

Figure S20. Single molecule fluorescence intensity Gaussian fitting. (A) Raw data for a typical single-molecule spot captured in a single image. The shown molecule is the median SSE value of all fits, meaning it is a good representative selection. The area to be fit is a 7x7 pixel image. (B) Fit for the raw data using Equation S2, where the variables which define the fitted surface are depicted. (C) Combined raw data and fitted surface.

attempting to fit two peaks to a single Gaussian, as seen in **Figure S9 C**. These molecules being close to each other would bias future analysis and decrease fidelity of the measurement, and are thus filtered out using their SE values as a metric for exclusion.

7.2 Analysis of SM Data 2: Obtaining time-traces of single-molecule events

The previous section identified optimal single-molecule events, but does not yet take into account the temporal nature of the experiment. Namely, if the same molecule is observed in 10 frames the previous analysis will identify it as 10 different ROIs when in reality all ROIs belong to the same molecule. We grouped individual ROIs into clusters of molecules using a weighted DBSCAN algorithm, where the weighting of the dimensions clustered molecules that were within 1.2 pixels in the x and y-spatial dimensions, and 5 frames in the temporal dimension (to account for rapid fluctuations in LR probe conformations, dye blinking, etc.). After clustering, the fits which did not successfully cluster were removed (~2.5%) as they represent either noise or molecules with fluorescent signal which only exist for a single time-frame (faster than our temporal resolution). Compiling all relative positions of successfully clustered molecules at the single-molecule level is shown in (**Figure S21 A,B**), where we selected a cluster composed of a single molecule existing across hundreds of frames and histogram the Gaussian peak's location in the x and y-dimensions. As can be seen, the Gaussian peak's position remains relatively stable across the registered time lapse, as expected. We then compared the position of the Gaussian peak's position (the *fit* center) with the pixel that defines its ROI center (the *raw data* center) across all molecules within a measurement (**Figure S21 C**), which shows the precision of our localization. Since the fluorescent LR probes are immobilized on a surface, the positional distributions are due to a combination of noise, camera drift, etc. and validate the 1.2 pixel parameter used in the DBSCAN clustering (all fits within a cluster are within 1.2 pixels of each other, even when comparing different clusters' relative positions with each other, (**Figure S21 C**).

After clustering fluorescence events into single-molecule localizations the positions of all events within a cluster were averaged together to find the most consistent central position of that molecule (e.g., averaging all points in **Figure S21 A** and **B** for that cluster). Finally, to create a time trace for that cluster, the intensities of the 3x3 area of pixels centered at the cluster's central position were summed for each frame across the timescale of the experiment to create an intensity time trace for that molecule (**Figure S22**).

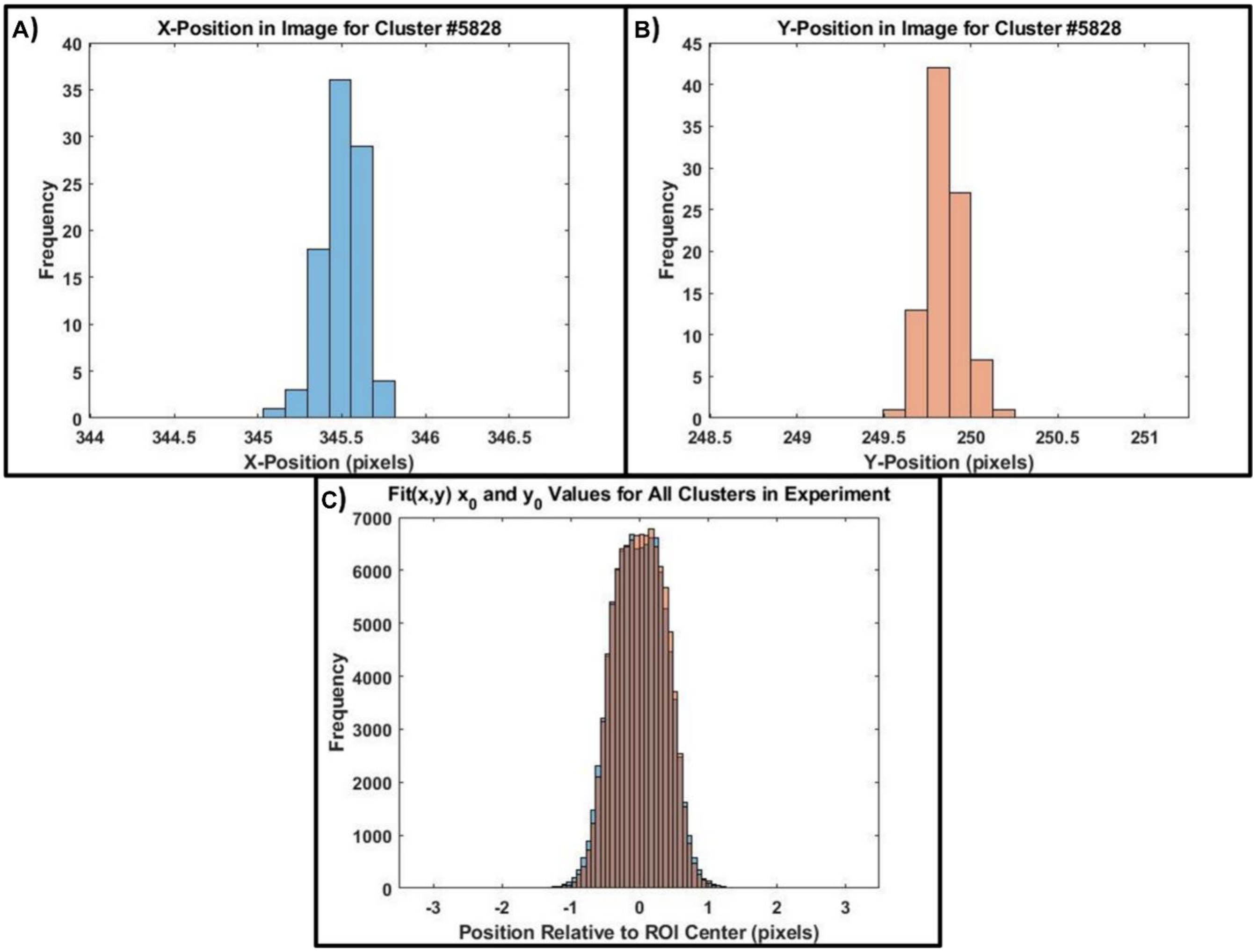

Figure S21. Distributions of the x_0 and y_0 values from fits using Equation S2, where all values reported passed through the filtering process described.

(A,B) The X and Y-Positions of a single cluster spanning multiple time frames (and therefore several fits) where all values are ascribed to an individual molecule. The x-axis shows the molecules absolute position on the images after registration. (C) The raw x_0 and y_0 positions for all fit molecules after filtering. The x-axis is relative to the center of the initial ROI obtained as described in this SI.

The time traces within **Figure S22** contain information on how each molecule is behaving during the timescale of the experiment. For instance, **Figure S22 B** (orange) shows a high intensity at the beginning of the experiment, suggesting that either the LR probe was opened *before* the image acquisition began (during the pre-incubation period while waiting for the cell to spread on the surface) or that the Cy3B dye for this probe began the experiment unquenched (due to the quencher photobleaching before data acquisition began or due to it not being labeled). In contrast, **Figure S22 C,D** show a probe where the Cy3B intensity starts low (quenched) and it becomes unquenched (most likely due to opening of the probe, but possibly due to infrequent photobleaching of the quencher). In all cases, the intensity goes up in a single step and decreases down to background in a single step, providing strong evidence that we are observing the LR probe dynamics at a single-molecule level.

Determining the timepoints when an intensity rises and falls, as well as the previously derived position of each single-molecule trace in both the donor and FRET channels is further analyzed in the next section, and the information is used determine kinetic information about the LR probe.

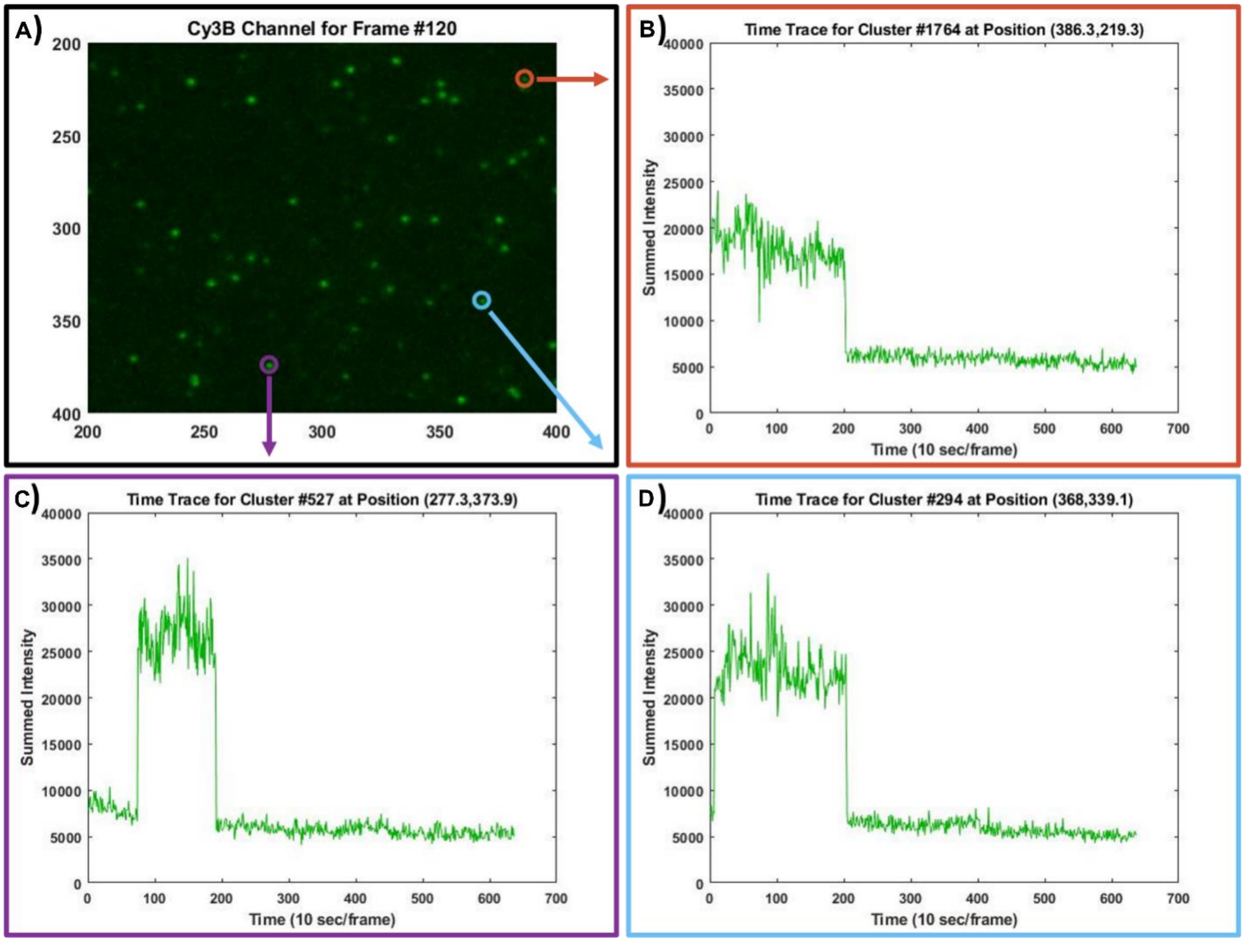

Figure S22. Time trace derivation from single-molecule events. (A) A 200x200 pixel zoom of the Cy3B channel at Frame 120 (20 minutes into the experiment). (B,C,D) Time traces for three randomly selected clusters, selected from the set of clusters centered within the 200x200 pixel range which were on during frame #120.

7.3 Analysis of SM Data 3: Identifying key timepoints within time-traces and sorting events

In order to quantify when intensity shifts happened within a probe we employed a changepoint algorithm[11]. We performed this algorithm on all single-molecule events while setting the maximum

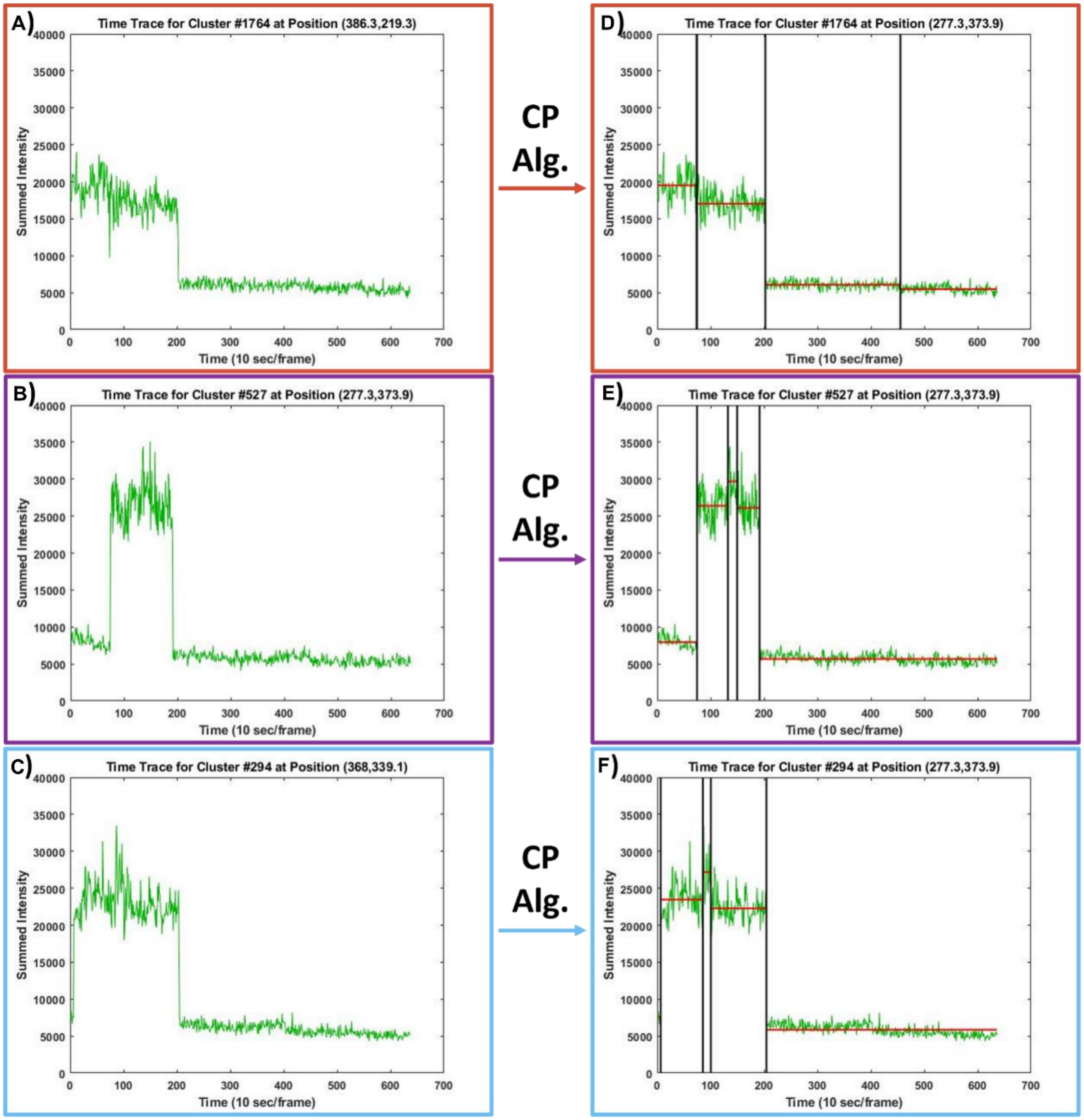

Figure S23. Single-molecule traces after implementing a change-point algorithm. (A,B,C) Initial single-molecule time traces reproduced from Figure S15. (D,E,F) Same traces after the change-point algorithm (CP Alg.) was applied. Black lines show the time points of each changepoint. Red lines show the average intensity value between changepoints.

number of intensity changes to 4 (off -> on -> off -> on -> off). This maximum number of changepoints was implemented to reduce the number of "false positives" due to the noise of single-molecule measurements, while still allowing the capture of all significant kinetic events within each single-molecule trace.

The same representative traces from **Figure S22** are reproduced in **Figure S23**, but with the before/after implementation of the changepoint algorithm (CP Alg.) visualized on top of the intensity traces. The timepoints for each changepoint are marked in black and, as can be seen, the CP algorithm has a tendency to "overfit" the intensity traces (**Figure S23 D,E,F**), which is why the constraint of a maximum of 4 changepoints was added. An example of this "overfitting" can be seen in **Figure S23 D**, where a changepoint is found at time frame ~450, where it detects a difference in background intensity. It should be noted that interesting fluorescent properties *may* be linked to these changepoints, and that these "overfit" changepoints are not necessarily false detections. However, our work is primarily interested in large intensity shifts resulting from probe opening/closing/rupturing events. To restrict our analysis to these events, it is beneficial to limit the maximum number of changepoints so only the largest intensity shifts are detected. In addition to finding the timepoints for each changepoint (black vertical lines), we also obtain the average intensity between changepoints (red horizontal lines). Taking the average intensity before/after each changepoint informs us on how significant the intensity shift is between detected changepoints, as well as the direction of those changepoints (on vs. off events).

7.4 Analysis of SM Data 4: Compiling kinetics of LR probe-rupturing event

Having analyzed the Cy3B (donor) channel to filter single-molecule events, fit their super-resolved positions, create intensity time traces, and obtain information on the intensity shifts as described above, the same analysis was repeated on the FRET channel to obtain two parallel data sets of the same dataset. A probe rupturing will follow the timeline of a probe opening (Cy3B turn-on) and the probe rupturing (Cy3B turn-off and FRET turn-on occurring simultaneously). We identified LR probe rupture events using the following criteria:

1) Two single-molecule cluster localizations in the Cy3B and FRET channels must be within 2 pixels of each other.

2) The Cy3B time trace must experience a turn-off event.

3) The FRET time trace must experience a turn-on event.

4) The turn-off/turn-on events in 2 and 3 must occur within a certain time of each other (threshold was within 2 frames).

For this representative dataset a total of 40 rupture events were found which satisfied the above criteria. 15 randomly selected unique traces are shown in **Figure S24** to represent the dataset. In these representative traces, the timepoints for probe opening (Cy3B turn-on) are shown in purple, while the average timepoints for criteria 2 and 3 (Cy3B turn-off, FRET turn-on) are shown in blue. The total amount of time the probe spent in the opened state before rupturing is the difference between the blue and purple timestamps (shown as ΔT in the figure titles in **Figure S24**), and has a time-resolution of 10 seconds (imaging framerate).

In addition to calculating the ΔT or probe-opening to probe-rupture, the time-traces also show additional evidence for single-molecule probes by analyzing the turn-off events. After rupturing, the probe has two dyes (donor and acceptor) which contribute to the FRET signal: if the donor photobleaches both

signals should go to background (**Figure S24 C,D,G,K,L,M,O**) or if the acceptor photobleaches the donor signal should increase while FRET signal goes to background (**Figure S24 B,E,F,H,J,N**) which may or may not be followed by donor signal photobleaching, if this occurs on the timescale of the experiment. These step-wise transitions provides even further evidence that the timescales we are observing come from true single-probe measurements.

Lasty, the locations and timepoints of these probe-rupturing events can be mapped in real-time onto the cell's position (as in **SI Video 1, 4-10**). These videos shows an RICM image of the cell taken in parallel with the donor and FRET channels. The red circles overlapped on top of the RICM show the positions of the clusters, and the frame in which the red circles appear correspond to time of probe rupturing (blue line, **Figure S24**). The video also contains a real-time summation of the total number of probe-rupture events occurring within the measurement, as well as the frame count. As is visually apparent from the video, the probe-rupture events mostly occur within the region occupied by the periphery of the cell, where tension is expected to be strongest, providing further strong evidence that these events are real and not due to noise inherent to single-molecule experiments.

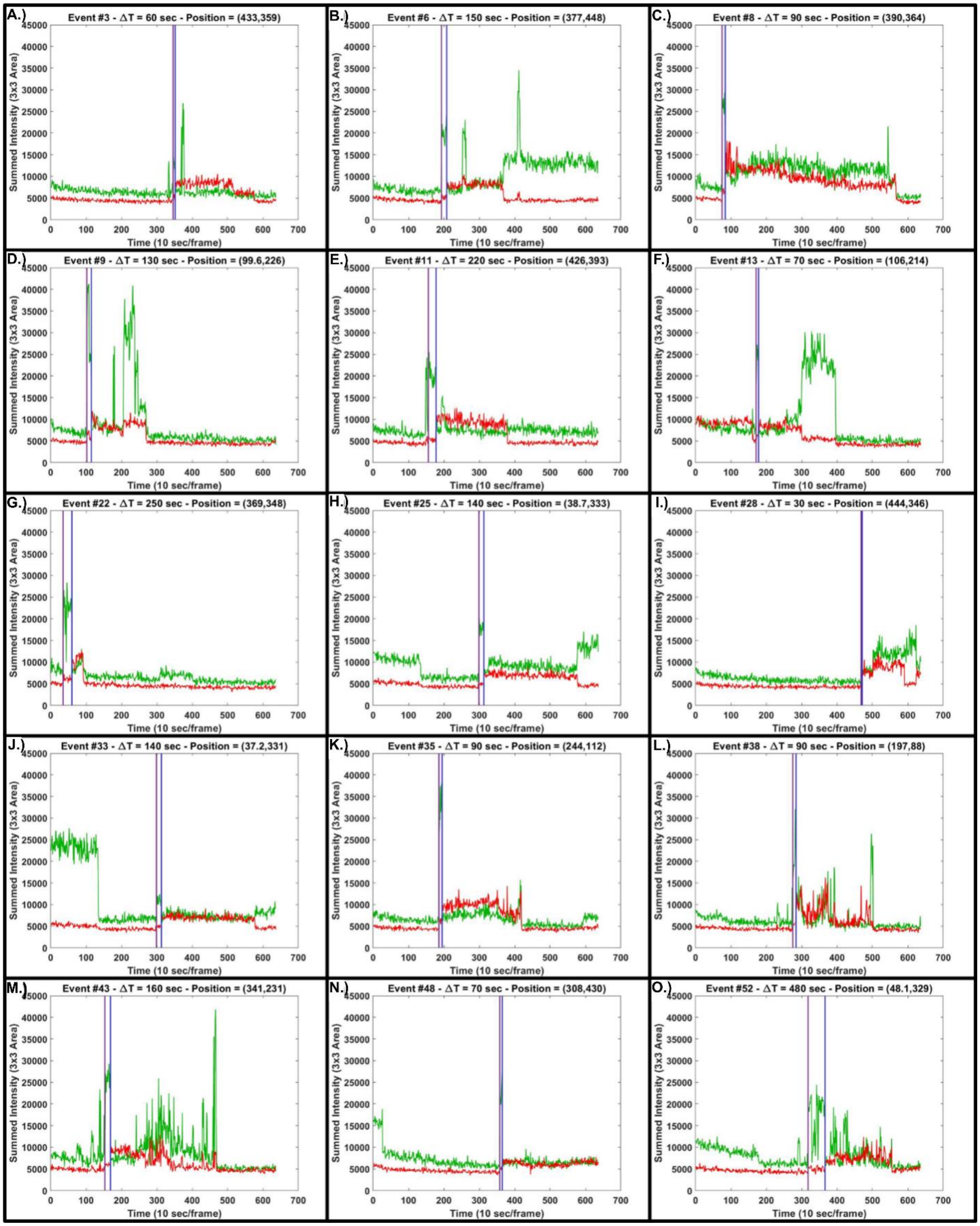

Figure S24. Time traces for Cy3B (green) and FRET (red) channels for rupture events, as identified by the 4 criteria listed above. The timestamp for the probe opening (Cy3B turn-on, purple) and the probe rupturing (Cy3B turn-off and FRET turn-on, blue) are shown as vertical lines. The cluster event index, rupture time (ΔT = blue – purple), and cluster position are shown in image titles.

7.5 Analysis of SM Data 5: Identifying Closed→Opened→Closed Transitions

Further events can be extrapolated from the data by setting a different set of criteria from those outlined in Section 4: Compiling kinetics of LR probe-rupturing event. For instance, identifying the probes which undergo a closed→opened→closed transition were found using the following criteria:

1) The donor signal must start at a signal close to background (low-signal state, for our experiments this was 7,000 counts across the 3x3 pixel region).

2) The signal must then increase in intensity by at least double (high-signal state)

3) Finally, the signal must then return to the low-signal state.

Additional selection criteria were applied to filter the events to make sure the signal came from a single molecule, further criteria included:

1) Transition events must not be within 2 pixels of each other (to avoid misinterpretation of overlapped of signal from single-molecule LR probes).

2) Event location must remain within camera FOV throughout the experiment (to remove false turn-on or turn-off signals due to camera drift during experiment).

For other experiments, additional criteria may be added are sensible within those parameters.

Lastly, for obtaining these timepoints and intensities of these transitions a changepoint algorithm was used, with a high error threshold set to find the most obvious changepoints. As the experiments can span a large timescale (sometimes > 600 frames), and a high-signal state can exist for as short as 1 frame, approximately 10% of events were so short-lived as to be missed by the algorithm with broad parameters. To compensate for this, a "second-pass" was performed on events which did not identify a high-signal state, and individual intensity points were identified which exceeded twice the intensity of their assigned

state. Such high-intensity points were assigned to the opened state, and the dataset of filtered closed→opened→closed transitions is represented in **Figure 4d**.

From **Figure 4d** and **SI Video 3**, it is clear that some events assigned closed→opened→closed transitions follow photobleaching kinetics (**Figure 4d**) and/or occur outside the area of the cell (**SI Video 3**). These events are likely due to photobleaching of the Q_1, which allows the donor to turn on, and results in a false "opened state". It is relatively simple to "background subtract" these photobleaching events, through the fits shown in **Figure 4d**: The relative area under each fit shows approximates the relative population for each event type (opened vs bleaching). Thus the final numerical values reported in the manuscript and represented in **Figure 4c** are the total number of events multiplied by the relative population of opened:bleach events (in our case, 77.4%).

SI Note 8 – Single-cell loading rate distributions

Another advantage of single-molecule spectroscopy is the ability to parse at the single-cell level which loading rate events are "fast" for a given cell, and which events are "slow". In **Figure 4a** of the manuscript we show the distribution of loading rates for all N = 138 shearing events across N = 8 cells. We can further parse those 138 events into the sub-distributions contributed by each cell, which is shown in **Figure S25** where the median loading rate for each cell is shown as a vertical, colored line.

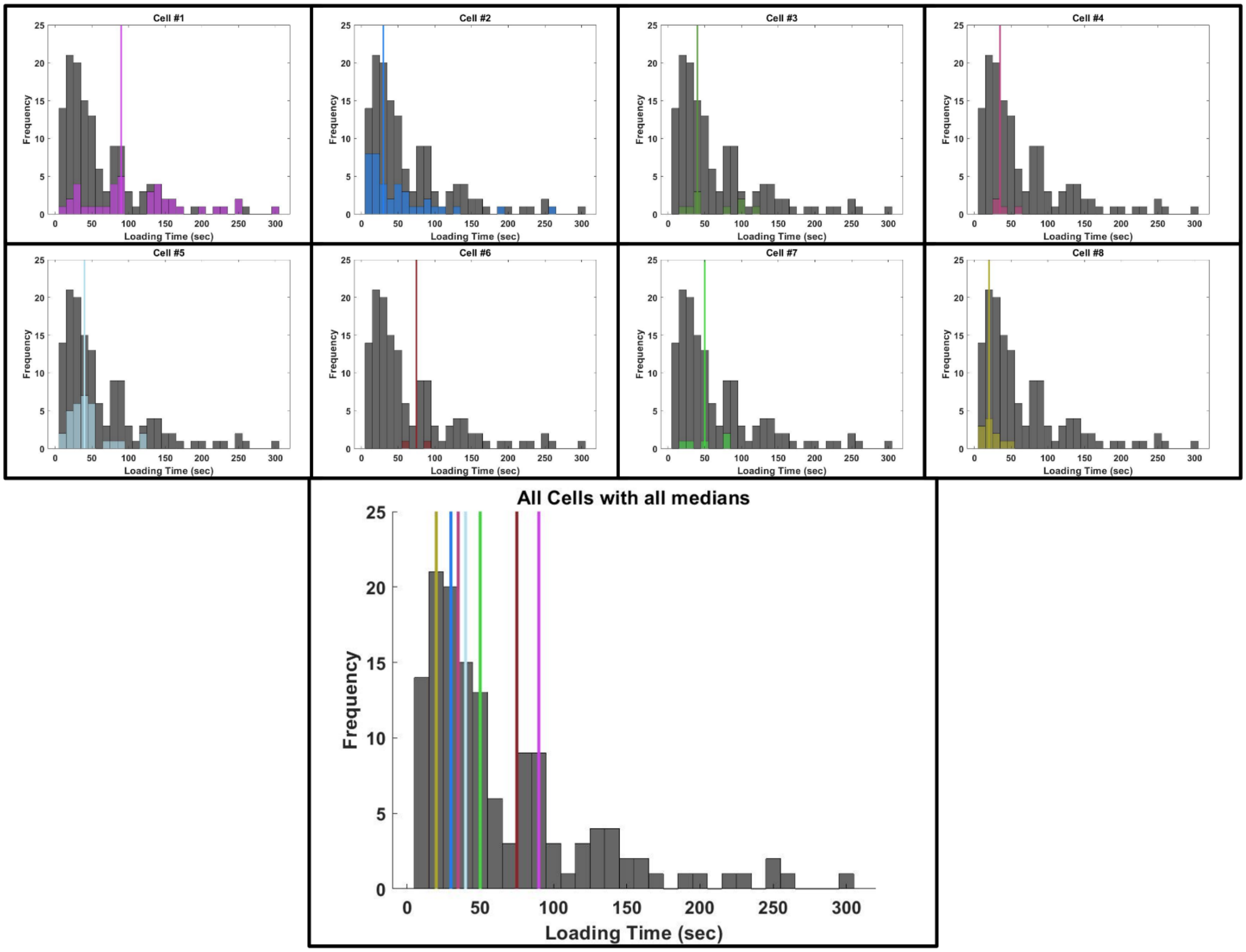

Figure S25. Top: The loading rate distribution for each cell, overlapped with the loading rate distribution from all cells combined. Vertical colored lines show the median value for the single-cell loading rate. **Bottom:** Loading rate distribution from all cells, with the various median values for the loading rates in their corresponding colors.

Once the median loading rate for each cell is determined (**Figure S25**), the cell's shearing events can be categorized as "fast" (≤median time) or "slow" (> median time) for that cell based on each event's loading rate. We trace the evolution of fast and slow events in **SI Video 11** for Cell #1 (the cell with the broadest distribution of loading rates), where the fast events are depicted as purple circles and the slow events are depicted as black circles in the video. We saw no obvious correlation between loading rate and the probe position, or between loading rate and the probe rupture time.

SI Note 9 – ESI-MS

The molecular weight of synthetic products was measured with an electro-spray ionization (ESI) method using a Thermo Fisher Scientific LTQ Orbitrap. The samples were prepared in the mixture of 70% 18.2 MΩ Nanopure water and 30% methanol containing 10 μM ethylenediaminetetraacetic acid (EDTA), 0.0375% triethylamine, and 0.75% of 1,1,1,3,3,3-hexafluoro-2-propanol (HFIP) and recorded the spectra with negative charge mode eluted with same solution. The main peak of obtained ESI-MS spectrum (m/z) was then deconvoluted to obtain average molecular weight for the oligonucleotides[18].

SI Note 10 – Manual and Picasso assisted analysis of single-molecule data

To qualitatively measure single-molecule fluorescence traces, a square 4X4 pixel (0.43X0.43 micron) square ROI was drawn around manually identified localizations. The average intensity of these localizations was measured over time to derive the intensity time traces. Only traces demonstrating single increase after acceptor photobleaching and single step decrease to background upon photo-bleach of Cy3B were used to calculate intensity values and FRET efficiency (**Figure S4**). Single molecule Cy3B brightness has been demonstrated to vary in fluorescence intensity of more than 20% for time traces on the order of minutes[7,8]. The FRET efficiency $E = \frac{I_{d,0}-I_d}{I_{d,0}-I_{d,BG}}$ was calculated where $I_{d,0}$ is the donor intensity after acceptor photobleaching, I_d is the donor intensity during FRET, $I_{d,BG}$ is the donor channel background intensity determined after donor photobleaching(**Figure S4 A**)[20]. The number of consecutive frames where Cy3B maintains high fluorescence after A647N acceptor photobleaching ($I_{d,0}$) was used to determine the average number of frames before Cy3B photobleaching, yielding an average of 71.1 frames, a standard deviation of 51.0 frames, and a standard error of the mean of 6.274 (**Figure S19 C**).

SI Note 11 – LR Probes Report Integrin-Ligand Tension

We set out to confirm the LR probe response to integrin mechanical engagement using NIH 3T3 fibroblasts (**Figure S26**). Because shearing is irreversible, the shearing signal (F>47 pN) is cumulative whereas the opening signal is only present during active (real-time) force application (**Figure S26 B-D**). To investigate if cells can open and shear LR probes, we incubated 3T3 cells on surfaces functionalized with LR probes for 45 minutes and compared the increase in intensity in the Cy3B, A647N direct excitation (A647N) and Cy3B (donor) sensitized A647N (acceptor) FRET channels (**Figure S26 B-D**). The enhancement in fluorescence (fold increase) signal in the sensitized FRET channel is comparable to the acceptor channel, while the fold increase in the Cy3B channel is much lower (**Figure S26 D**). This is consistent with the model that A647N and FRET report a cumulative history of irreversible shearing events while Cy3B reports reversable real-time force events which should be less abundant. Opening signal is also largely localized with the cell RICM edge (**Figure S26 B**) as found in previous studies of fibroblasts spreading on hairpin probes within the first hour after plating[1]. Likewise in agreement with previous studies on 47 pN TGTs shearing signal is apparent throughout the body of the cell (**Figure S26 B**) where strong integrin forces ruptured probes throughout the spreading process[2].

To test the LR probe under single molecule imaging conditions, we first had to reduce the density of the probes to appropriate concentrations. The Cy3B background signal demonstrates a false positive on-rate of 22% for LR probe surfaces functionalized with 0.8 Cy3B and A647N functionalized probes per µm2 (**Figure S3**). This is likely due to a combination of BHQ2 quencher photobleaching/oxidative degradation, spontaneous top strand dissociation with acceptor photobleaching, and hairpin breathing.[3,4] Cy3B intensity has a high variability of 10±3% of its average intensity for single stranded probes after acceptor photobleaching and 14±13% of its average intensity for opened hairpins with locking strand (**Figure S5 A**), in rough agreement with in previous studies.[5,6] Single molecule Cy3B brightness has been

demonstrated to vary in more than 20% for time traces on the order of minutes.[7,8] The high variability of Cy3B fluorescence intensity is likely due to uneven TIRF field illumination and other photo-physical factors. We empirically titrated different ratios of LR probe to find a concentration which provided sufficient surface functionalization to detect single molecule events and found that exposing surfaces to between 0.53 and 0.26 nM annealed LR probe provided minimal presence of un-quenched Cy3B localizations while confirming the presence of surface functionalization as measured by the Picasso software package (**Figure S3**).[9]

To test whether ligand-selective opening signal can be detected from cells at low probe density we took timelapse micrographs with a 10 sec frame interval of 3T3 cells plated on surfaces with low density of LR probe supplemented with cRGD-dsDNA-biotin(**Figure S2**, **S10A**). Because the opening signal (Cy3B) has a 20% false positive Cy3B on-signal (**Figure S28**) in areas not associated with the RICM cell area, we compared these cells to cells plated on surfaces functionalized with LR probes lacking cRGD ligands (**Figure S27 B**). To visualize Cy3B and FRET turn-on events we derived images of the maximum intensity of each pixel which occurs throughout the timelapse micrograph and binned the resulting image (**Figure S27 A insets**). For cells plated on LR probes functionalized with cRGD ligands, there is an increase in intensity in the Cy3B and FRET channels within the RICM cell area relative to background area outside the cell, that is more evident with image binning (**Figure S27 A**). For cells plated on LR probes lacking cRGD ligands, the distribution of un-quenched Cy3B, due to quencher photo-bleaching and hairpin breathing, is evenly distributed throughout the field of view (**Figure S27 B**). The frequent opening of probes by cells can be visualized by the difference image, derived by subtracting the Cy3B and FRET fluorescence micrographs of the previous frame from the current frame, within the RICM area under the cell (**Figure S27 C**). This, along with the maximum intensity image, corroborates that Cy3B turn on events

occur more frequently for LR probes with ligands in contact with the cell (RICM) due to integrin-ligand force.

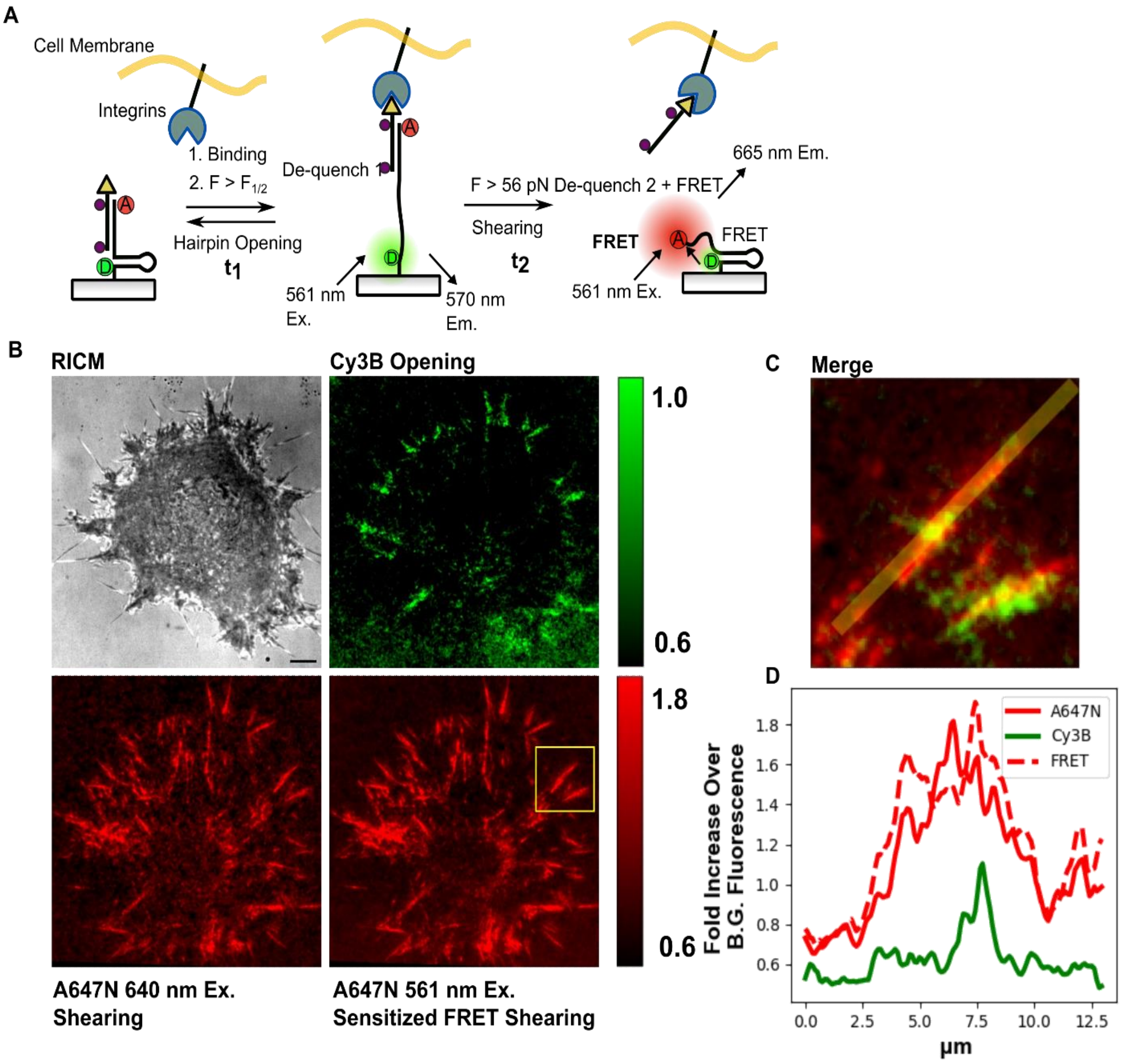

Figure S26. Loading rate probes report cell tension in Cy3B, A647N (640 nm excitation), and sensitized FRET fluorescence channels. (A) Diagram of loading rate probe dual force threshold fluorescence readout process. (B) Reflection interference contrast microscopy (RICM), Cy3B, A647N, and A647N excited by Cy3B sensitized FRET fluorescence micrographs of a 3T3 Cell plated on for 45 min on surfaces exposed to a solution of 6.7 nM biotinylated LR probes for 45 min., scale bar 5 µm, calibration bar Fold increase over background fluorescence (measured by dividing the cell micrograph by a micrograph of the same surface in an adjacent location lacking the cell). (C) Merge of fluorescence channels in yellow inset region in B with linear ROI. (D) Fold increase over background fluorescence of yellow line-scan in C demonstrating increased intensity of A647N and FRET over Cy3B due to irreversible shearing contrasted with reversible hairpin opening.

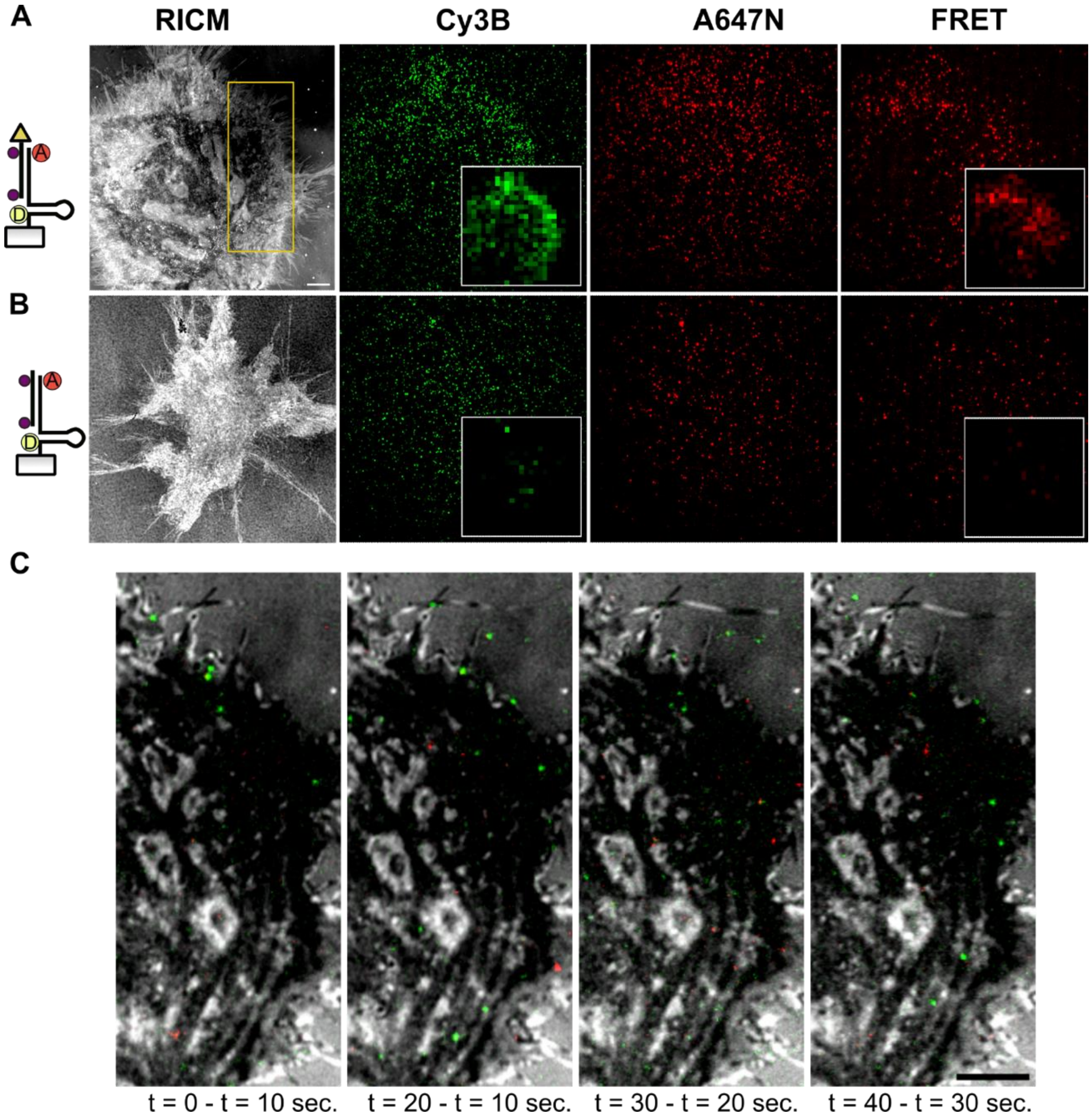

Figure S27. LR probes report ligand specific opening and shearing at low probe density. Cumulative maximum intensity images derived by taking the maximum of each pixel in 90 minute timelapse fluorescence and RICM micrographs of (A) 3T3 cells plated for 40-60 min. on surfaces exposed to 0.40 nM biotinylated LR probe for 45 min flowed by 6 nM cRGD-dsDNA-biotin for an additional 45 min. (B) Surfaces exposed to LR probe lacking cRGD under the same conditions, both with 20x20 pixel average binning of the Cy3B and FRET image insets showing increased intensity for cRGD ligand presenting probes. (C) Inset outlined in yellow of A, RICM micrographs(greyscale) overlaid with Cy3B(green) channel difference images, produced by subtracting the previous Cy3B fluorescent micrograph frame in the timelapse from the subsequent frame, demonstrating newly opened hairpins by cells and minimal background false positive opening events.

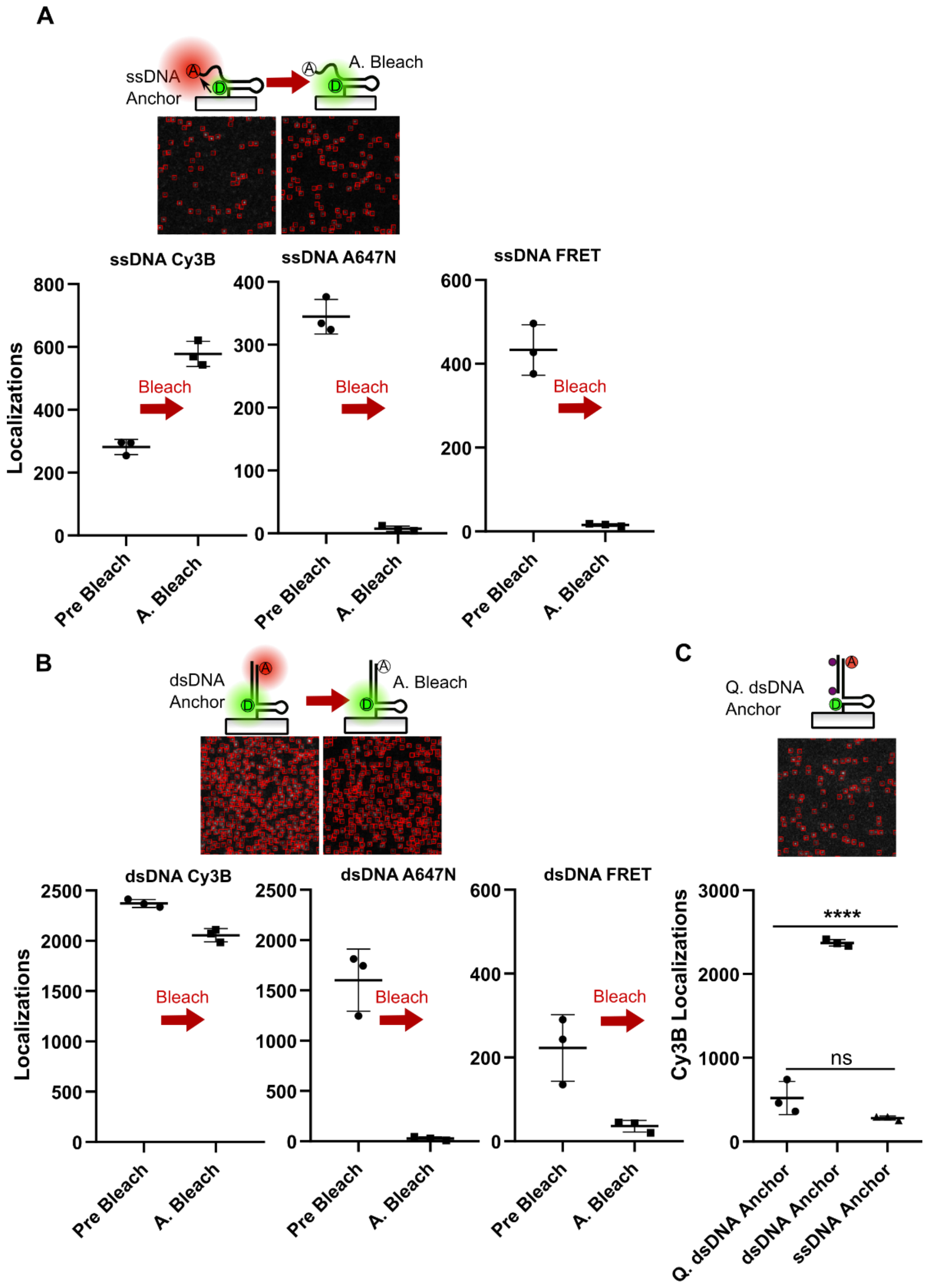

Figure S28. Localizations per FOV (2982 μm^2) of different LR probe anchor constructs. Diagram of ssDNA Anchor, dsDNA anchor, and quenched Q. dsDNA anchor constructs and representative micrographs of biotinylated surfaces exposed to 0.4 nM of the (A) single stranded DNA

LR probe anchor and (B) dsDNA LR probe anchor hybridized to the ligand domain complimentary strand lacking a ligand or quenchers with plots of Localizations in A647N channel Cy3B channel and FRET channel before and after accepter photobleaching demonstrating an increase in Cy3B localization after photobleaching for ssDNA anchors and a decrease for dsDNA anchors with Picasso localizations of Cy3B boxed in red before and after photobleaching of the A647N FRET acceptor. Micrographs are 54.61X54.61 µm. (C) Cy3B localizations including quenched dsDNA LR anchor demonstrating an average 521±196 bright Cy3B localizations for quenched LR probe anchors, 2372±39 localizations for non-quenched dsDNA anchors, and 281±23 localization for ssDNA anchors demonstrating the fraction of false-positive background Cy3B upon photobleaching.

References

(1) Zhang, Y.; Ge, C.; Zhu, C.; Salaita, K. DNA-Based Digital Tension Probes Reveal Integrin Forces during Early Cell Adhesion. *Nat. Commun.* **2014**, *5*, 5167. https://doi.org/10.1038/ncomms6167.

(2) Wang, X.; Sun, J.; Xu, Q.; Chowdhury, F.; Roein-Peikar, M.; Wang, Y.; Ha, T. Integrin Molecular Tension within Motile Focal Adhesions. *Biophys. J.* **2015**, *109* (11), 2259–2267. https://doi.org/10.1016/j.bpj.2015.10.029.

(3) Peyrard, M.; Cuesta-López, S.; James, G. Nonlinear Analysis of the Dynamics of DNA Breathing. *J. Biol. Phys.* **2009**, *35* (1), 73–89. https://doi.org/10.1007/s10867-009-9127-2.

(4) Ryabinin, V. A.; Kostina, E. V.; Sinyakov, A. N. Unexpected Transformation of Black Hole Quenchers in Electrophoretic Purification of the Fluorescein-Containing TaqMan Probes. *Nucleosides. Nucleotides Nucleic Acids* **2017**, *36* (6), 418–427. https://doi.org/10.1080/15257770.2017.1310384.

(5) Ambrose, B.; Baxter, J. M.; Cully, J.; Willmott, M.; Steele, E. M.; Bateman, B. C.; Martin-Fernandez, M. L.; Cadby, A.; Shewring, J.; Aaldering, M.; Craggs, T. D. The SmfBox Is an Open-Source Platform for Single-Molecule FRET. *Nat. Commun.* **2020**, *11* (1), 5641. https://doi.org/10.1038/s41467-020-19468-4.

(6) Le Reste, L.; Hohlbein, J.; Gryte, K.; Kapanidis, A. N. Characterization of Dark Quencher Chromophores as Nonfluorescent Acceptors for Single-Molecule FRET. *Biophys. J.* **2012**, *102* (11), 2658–2668. https://doi.org/10.1016/j.bpj.2012.04.028.

(7) Patra, S.; Claude, J. B.; Wenger, J. Fluorescence Brightness, Photostability, and Energy Transfer Enhancement of Immobilized Single Molecules in Zero-Mode Waveguide Nanoapertures. *ACS Photonics* **2022**, *9* (6), 2109–2118. https://doi.org/10.1021/acsphotonics.2c00349.

(8) Plochowietz, A.; Crawford, R.; Kapanidis, A. N. Characterization of Organic Fluorophores for in Vivo FRET Studies Based on Electroporated Molecules. *Phys. Chem. Chem. Phys.* **2014**, *16* (25), 12688–12694. https://doi.org/10.1039/c4cp00995a.

(9) Schnitzbauer, J.; Strauss, M. T.; Schlichthaerle, T.; Schueder, F.; Jungmann, R. Super-Resolution Microscopy with DNA-PAINT. *Nat. Protoc.* **2017**, *12* (6), 1198–1228. https://doi.org/10.1038/nprot.2017.024.

(10) Maus, M.; Cotlet, M.; Hofkens, J.; Gensch, T.; Schryver, F. C. De. An Experimental Comparison of the Maximum Likelihood Estimation and Nonlinear Least-Squares Fluorescence Lifetime Analysis of Single Molecules. *Anal. Chem.* **2001**, *73* (9), 2078–2086.

(11) Killick, R.; Fearnhead, P.; Eckley, I. A. Optimal Detection of Changepoints with a Linear Computational Cost. *Journal of the American Statistical Association*. 2012, pp 1590–1598. https://doi.org/10.1080/01621459.2012.737745.

(12) Wang, X.; Ha, T. Defining Single Molecular Forces Required to Activate Integrin and Notch Signaling. *Science (80-.).* **2013**, *340* (6135), 991–994. https://doi.org/10.1126/science.1231041.

(13) Snodin, B. E. K.; Randisi, F.; Mosayebi, M.; Šulc, P.; Schreck, J. S.; Romano, F.; Ouldridge, T. E.; Tsukanov, R.; Nir, E.; Louis, A. A.; Doye, J. P. K. Introducing Improved Structural Properties and Salt Dependence into a Coarse-Grained Model of DNA. *J. Chem. Phys.* **2015**, *142* (23), 234901. https://doi.org/10.1063/1.4921957.

(14) Mosayebi, M.; Louis, A. A.; Doye, J. P. K.; Ouldridge, T. E. Force-Induced Rupture of a DNA Duplex: From Fundamentals to Force Sensors. *ACS Nano* **2015**, *9* (12), 11993–12003. https://doi.org/10.1021/acsnano.5b04726.

(15) Poppleton, E.; Bohlin, J.; Matthies, M.; Sharma, S.; Zhang, F.; Šulc, P. Design, Optimization and Analysis of Large DNA and RNA Nanostructures through Interactive Visualization, Editing and

Molecular Simulation. *Nucleic Acids Res.* **2020**, *48* (12), E72–E72. https://doi.org/10.1093/nar/gkaa417.

(16) Hatch, K.; Danilowicz, C.; Coljee, V.; Prentiss, M. Demonstration That the Shear Force Required to Separate Short Double-Stranded DNA Does Not Increase Significantly with Sequence Length for Sequences Longer than 25 Base Pairs. *Phys. Rev. E* **2008**, *78* (1), 011920. https://doi.org/10.1103/PhysRevE.78.011920.

(17) Engel, M. C.; Smith, D. M.; Jobst, M. A.; Sajfutdinow, M.; Liedl, T.; Romano, F.; Rovigatti, L.; Louis, A. A.; Doye, J. P. K. Force-Induced Unravelling of DNA Origami. *ACS Nano* **2018**, *12* (7), 6734–6747. https://doi.org/10.1021/acsnano.8b01844.

(18) Hail, M. E.; Elliott, B.; Anderson, K. High-Throughput Analysis of Oligonucleotides Using Automated Electrospray Ionization Mass Spectrometry. *Am. Biotechnol. Lab.* **2004**.

(19) Preibisch, S.; Saalfeld, S.; Schindelin, J.; Tomancak, P. Software for Bead-Based Registration of Selective Plane Illumination Microscopy Data. *Nat. Methods* **2010**, *7* (6), 418–419. https://doi.org/10.1038/nmeth0610-418.

(20) Chang, A. C.; Mekhdjian, A. H.; Morimatsu, M.; Denisin, A. K.; Pruitt, B. L.; Dunn, A. R. Single Molecule Force Measurements in Living Cells Reveal a Minimally Tensioned Integrin State. *ACS Nano* **2016**, *10* (12), 10745–10752. https://doi.org/10.1021/acsnano.6b03314.

(21) Liu, J.; Yan, J. Unraveling the Dual-Stretch-Mode Impact on Tension Gauge Tethers' Mechanical Stability. *J. Am. Chem. Soc.* **2024**, *146* (11), 7266–7273. https://doi.org/10.1021/jacs.3c10923.
